# Changes in Berry Tissues in Monastrell Grapevines Grafted on Different Rootstocks and Their Relationship with Berry and Wine Phenolic Content

**DOI:** 10.3390/plants10122585

**Published:** 2021-11-25

**Authors:** Josefa M. Navarro, Pablo Botía, Pascual Romero

**Affiliations:** Irrigation and Stress Physiology Group, Murcia Institute of Agricultural and Environmental Research and Development, 30150 Murcia, Spain; pablo.botia@carm.es (P.B.); pascual.romero@carm.es (P.R.)

**Keywords:** phenolic compounds, anthocyanins, berry quality, regulated deficit irrigation, Monastrell vines, rootstocks

## Abstract

Monastrell grapevines grafted on the rootstocks 140Ru, 1103P, 41B, 110R, and 161-49C were subjected to regulated deficit irrigation (RDI) and partial root-zone irrigation (PRI). We analyzed the effects of the rootstock and irrigation method on the phenolic concentration in different berry tissues, its dilution/concentration due to the berry size, the anatomical and morphological traits of berries related to the phenolic compounds concentration, and the relationships of all these parameters with the final berry and wine phenolic content. The rootstock had an important effect on the accumulation of total phenolic compounds and anthocyanins in the skin (berries from 110R and 140Ru had the highest values). Moreover, the rootstock modified some anatomical and morphological characteristics that had a direct relationship with the final phenolic compounds concentration in the must. Large grapes and high must percentages (110R and 140Ru) produced a dilution effect, whereas small berries and a low must percentage increased the concentration (161-49C). For 110R, the small size of the cells of the epidermis and hypodermis in the grapes also could have contributed to the high phenolic compounds concentration in the skin. The percentage of cells in the skin with a uniform coloration was positively correlated with its total phenolic compounds and anthocyanins concentration and also with the phenolic quality of the wine. The PRI modified some specific morphological/anatomical skin/berry traits, and these may have contributed to important changes in the final concentration of phenolic compounds, depending on the rootstock. The better phenolic quality of the must and wines observed in some rootstocks under PRI could be due to smaller cells in the epidermis and hypodermis of the skin (161-49C), a higher percentage of cells with a uniform coloration in the hypodermis (110R), or a lower number of seeds per berry (161-49C). In contrast, the lower phenolic compounds concentration in the must of grapes observed in the most vigorous rootstocks under PRI could be due to a greater thickness of the epidermis (140Ru), greater cuticle thickness (41B), a higher number of seeds (140Ru), a lower skin/pulp ratio and percentage of skin (140Ru), a greater percentage of cells in the epidermis without coloration or with large inclusions, and a lower percentage of cells with a uniform coloration in the epidermis (140Ru). The final quality of the grape is related to some changes in histological and morphological aspects of the grape produced by the rootstock and irrigation strategy.

## 1. Introduction

Monastrell (Mourvedre in France, and Mataro in Australia and California) is an ancient, native, black-skinned grape variety originating from the Spanish Levante (southeastern, Spain) that has been grown in vineyards all around the western Mediterranean countries for centuries. Its cultivation now extends throughout Spain, southern France, California, and South Australia. Currently, this variety is grown principally in semiarid areas of the southeast of Spain, covering 43,000 ha (4.4% of the total vineyard area in Spain [1]), since it is well adapted to these rigorous and dry climates of high temperatures and recurrent drought cycles. In this sense, certain physiological characteristics of tolerance to drought have been identified in Monastrell, which give it a good capacity to adapt to the lack of water and to water stress [2,3].

Nowadays, due to new agronomic practices and winemaking techniques, this variety has an important oenological potential, as shown by the high-quality wines produced in D.O. Jumilla and D.O. Bullas (southern Spain), the increasing exports of these wines to other countries, and the high price/classification reached in the wine market by the wines created with Monastrell grapes. However, the climate change predicted for these semiarid zones, where there is already limited water availability, means that the climate will become warmer and drier [4], threatening vineyard sustainability in the near future. Thus, adaptation measures are necessary, especially those related to the application of deficit irrigation, improvements in water use efficiency, and the selection of plant material (rootstocks, clones, new crosses/varieties of Monastrell) better adapted to the new climatic situation [5,6,7,8].

Genotypic differences in the vigor of the rootstocks induce morphological and anatomical modifications and changes in the distribution of the root system that can affect the regulation of water relations [9,10,11], yield components, and fruit and wine composition [12,13,14].

In previous studies in Monastrell vineyards under semiarid conditions, a synergic approach—with tolerant rootstocks (R) and well-designed deficit irrigation strategies/methods (IS: regulated deficit irrigation (RDI) and partial root-zone drying irrigation (PRI)) with low water volumes—produced substantial changes in the final berry quality and the chemical/aromatic composition and organoleptic perception of the wine, as well as in its concentration of compounds considered as nutraceuticals [15,16]. We hypothesize that most of the differences observed in the final wine composition and global quality were initially induced by changes in the berry (at the whole berry level and in the different tissues (seeds, pulp, and skins)) that were later manifested in the must and wine. These alterations may include berry tissue-specific changes in hormone signaling-induced gene expression and related metabolomic changes in the berry [17,18], as well as changes in the berry size and in the morphology, anatomy, and structure of berry tissues such as the skin, seed, cuticle, and cell wall [19,20,21]. Although skins represent a small percentage of the total berry weight, they are fundamental in wine quality, since most of the aromatic and phenolic compounds are located therein [21]. Therefore, it is necessary to know the structural/anatomical properties of the skins, since these can determine the mechanical resistance and texture of berries and affect the extraction of phenolic compounds from the skins to the must during the maceration process [21]. In particular, the skin thickness (which is genetically influenced) is one of the most important grape skin morphological characteristics and is a useful indicator of anthocyanin extractability, as thinner skins seem to be characterized by a higher anthocyanin extractability [22]. The skin/berry ratio is also a fundamental value to take into account during winemaking, since the higher the ratio, the greater the amount of the compounds of potential interest synthesized in the skin which can be released during the maceration process [23]. In addition, the cell walls are also responsible for several characteristics of the berries, including firmness and mechanical properties. Moreover, they may also act as a barrier to the extraction of phenolic compounds during winemaking [23,24]. Therefore, the study of skins is of great relevance regarding the degree of extraction of phenolics from the grapes, their transfer into the must and wine, and, thus, the wine quality. Nevertheless, the concentration/composition in the grape is not always reflected in the final wine concentration/composition, since several factors are involved both in the extraction of phenolic compounds from the grapes and in the processes of fermentation and maceration.

The skin of Monastrell grapes has more layers of cells and thicker cell walls than that of other varieties [20], which could explain the high extractability (EA) index of this variety. Most of the skin flavonoids are located in the thick-walled inner layers of the hypodermis. In addition, Monastrell berries have thicker skins and a higher cellulose content in the cell walls than those of other varieties [25], which may explain the difficulties usually observed in the extraction of anthocyanins from Monastrell grapes during winemaking [23,24]. Changes in seed maturity and the seed tannin content are also important, because they may help to alter the seed/skin tannins ratio, affecting organoleptic attributes in the wine such as astringency and bitterness [26]. Thus, the study of different berry compartments (such as the skins and seeds) is important, as they are highly complex and dynamic components affected by factors such as the variety/rootstock, terroir-environmental conditions, microclimate, and irrigation [19,24,27]. The main objective of this study is to determine if the choice of the rootstock and the irrigation method affects berry tissues and how these anatomical and morphological changes are related to the phenolic content of the berry, must, and wine. Thus, the specific objectives of this study are:

(1) To analyze the changes in the accumulation of secondary metabolites (polyphenols) in the different berry tissues (skins and seeds) due to the rootstock, irrigation method, and their interaction.
(2) To study the anatomical/morphological changes in skins and seeds due to the rootstock and irrigation method.
(3) To study the concentration/dilution effects of changes in the berry size/weight and the skin/pulp/berry ratio, and their influence on the final must and wine polyphenolics concentrations.
(4) To look for significant relationships between berry tissue morphological, anatomical, and metabolomic changes and the final must and wine composition.

## 2. Materials and Methods

### 2.1. Field Conditions, Plant Materials, and Irrigation Treatments

This research was carried out from 2012 to 2016 in a 0.4 ha vineyard at the IMIDA experimental station in Cehegín, Murcia, SE Spain (38°6′38.13″ N, 1°40′50.41″ W, 432 m a. s. l.). The environmental and experimental conditions are detailed in previous studies [15]. 

The grapevines (*Vitis vinifera* L., var. Monastrell, syn. Mourvedre), grafted on five different commercial rootstocks (140Ru, 1103P, 41B, 161-49C, and 110R), were drip-irrigated during five consecutive years, using two different deficit irrigation (DI) strategies: RDI and PRI. Water was applied with one drip-irrigation line per row for the conventional drip irrigation in RDI, and using a double line per row for the PRI in which the emitters had double the flow. In this way, the same amount of water was applied in PRI and RDI. In the PRI layout, the two pipelines were joined on both sides of the trunk and were located underneath each vine row. In each pipeline in the PRI treatments, there were alternate zones with and without emitters to create wet and dry root zones within each vine row. In the PRI treatments, water was supplied to only one side of the root system at a time, alternating every 14–16 days. In the RDI treatments, irrigation water was supplied simultaneously to the entire root system. Similar annual water volumes were applied for irrigation in all the rootstock × irrigation strategy combinations [15]. The same tailored DI strategy was applied in all cases: (1) no or slight stress from budburst to fruit set; (2) moderate–severe water stress from fruit set to veraison; (3) partial irrigation recovery to maintain moderate stress from veraison to harvest; (4) full recovery postharvest.

### 2.2. Physicochemical Determinations in Grapes

Samples of mature berries were collected from each grapevine in September in the years 2012 to 2016, when the maturity was around 23–24° Brix (coinciding with harvest), and transported to the laboratory. The harvest dates were determined on the basis of weekly analyses of the grape composition.

The samples consisted of 800–900 g of berries collected randomly from different clusters on each vine. Sub-samples of 10 berries were weighed and separated for the determination of total phenolic compounds and total anthocyanins in the skin and seeds. The rest of the berries were crushed by an automatic blender, centrifuged, and analyzed for berry quality as described previously [15]. The berry phenolic quality index (QI_phenolic berry_) was also calculated, as described by Romero et al. [28]. Briefly, QI_phenolic berry_ was calculated classifying some important phenolic parameters into four groups according to their composition. Each group was given a value between 0 and 3: group 1, with the lowest score (0), had the worst composition and lowest quality and group 3, with the highest score (3), had the best composition and highest quality of grapes [28]. According to this classification, QI_phenolic berry_ was calculated using the following equation:

QI_phenolic berry_ = Ant_tot_ + Polyph_extr_ + A_520_ + berry weight + SM

where Polyph_extr_ is the extractable polyphenols, A_520_ is the absorbance at 520 nm, and SM is the seed maturity index. 

After centrifugation of the must at 4500 rpm for 20 min, the color intensity (CI) was calculated as the sum of the absorbances at 620 nm, 520 nm, and 420 nm. The tone was calculated as the ratio of the absorbance at 420 nm to that at 520 nm. A CR-10 colorimeter was used for CIELab parameter determination, recording the L* (lightness), a* (redness–greenness), and b* (yellowness–blueness) by measurement of the transmittance of the must every 10 nm from 380 to 770 nm, using the D65/10° for the illuminant/observer, in glass cells having a 0.2 cm path length. The chroma (C*) and hue angle (h*) were calculated as: C* = (a*^2^ + b*^2^)^½^ and h* = (tan^−1^ b*/a*). The chroma signifies the color purity or color intensity from achromatic (gray) towards chromatic color, starting from zero without any possible end point, although the intensity increased in magnitude. The hue angle refers to the color wheel (i.e., green, yellow, and red, with values of 180, 90, and 0, respectively) [29]. 

At the harvest in 2015, samples of 30 berries from different plots were collected for each rootstock and irrigation method, for analysis of the texture. For each berry, the skin hardness was assessed by a puncture test carried out on the equatorial area of the berry with a Texture Analyser Machine (MARK-10 Model BG 50 Force Gauge, Copiague, NY, USA).

### 2.3. Determination of Sugars in Grapes

A sample of 0.5 g of crushed grapes was extracted with 80% ethanol three consecutive times for sugars extraction, as has been previously described for sugar extraction in plant materials [30]. The ethanolic extracts were evaporated to dryness and each residue was dissolved in deionized water and filtered through a Sep-Pak C18 cartridge. Glucose, fructose, and sucrose were analyzed by high-performance anion-exchange chromatography with pulsed amperometric detection (HPAE-PAD), using Thermo Scientific Dionex CarboPac PA20 guard (3 × 30 mm) and separation (3 × 150 mm) columns. The eluent was 10 mM NaOH (isocratic) with a step to 200 mM KOH at 10 min to regenerate the column. The residue obtained after the ethanolic extraction was used for starch determination. Amyloglucosidase from *Aspergillus niger* was used for starch hydrolysis and the glucose units were analyzed as described for the soluble sugars.

### 2.4. Determination of the Total Phenolic Compounds and Total Anthocyanins in the Skin and Seeds

The standard phenotyping protocol proposed by Rustioni et al. [31] was adopted for eno-carpological evaluation of grapes. Briefly, at harvest time, 10 mature and representative berries were selected and peeled with a scalpel to separate the skins, pulp, and seeds. The fresh skins were weighed and the seeds were counted and weighed. The skins and seeds were then extracted in 20 mL of an ethanol:water:hydrochloric acid (70:29:1) solution for 24 h. The extracts were filtered before spectrophotometric determination of the total phenolics compounds by the Folin–Ciocalteu method. An aliquot (0.5 mL) of each extract (diluted appropriately) was added to a 10 mL volumetric flask containing 2.5 mL of distilled water. Then, Folin–Ciocalteu reagent (0.5 mL) was added and the contents mixed. After 3–5 min, 2 mL of a 10% Na_2_CO_3_ solution was added and the total volume was made up to 10 mL with distilled water. After keeping the samples for 90 min at room temperature, their absorbance was read at 700 nm (A_700_). The total phenolic compounds were expressed as the catechin concentration (mg L^−1^), calculated by applying the formula:

$${\mathrm{Total}\;\mathrm{phenolic}\;\mathrm{compounds}\;(\mathrm{mg}\;L}^{-1}{)=A}_{700}\;\times \;186.5$$

The total anthocyanins content in the skin extracts was calculated by converting the absorbance reading at 540 nm (A_540_) to the equivalent content of malvidin-3-*O*-glucoside, by multiplying it by the coefficient 16.17:

$${\mathrm{Total}\;\mathrm{anthocyanins}\;(\mathrm{mg}\;L}^{-1}{)=A}_{540}\;\times \;16.17$$

### 2.5. Histological Analysis of the Grapes

For the histological characterization, two days before the harvest in 2015, 40 mature, healthy, and representative berries from different plots of the orchard were randomly selected and collected for each R × IS combination and taken to the laboratory. The histological study was carried out according to the methodology described by Ortega-Regules et al. [32]. Briefly, fractions of grape skins (epidermis and hypodermis) from different berries were cut with a scalpel and immediately fixed in a solution of 3% glutaraldehyde (Ted Pella Inc., Redding, CA, USA) diluted with 0.1 mol L^−1^ sodium cacodylate buffer solution at pH 7.4, overnight at 4 °C. The samples were post-fixed in 1% osmium tetroxide for 2.5 h at 4 °C and then washed with buffer three times. After that, they were dehydrated in a graded ethanol series (30–100%) embedded in an epoxy resin (pure Spurr) (Sigma-Aldrich, St. Louis, MO, USA), and then left in an oven at 70 °C for 48 h. Sections of different thicknesses were cut using a Leica UC6 ultra-microtome (www.leica-microsystems.com, accessed on 17 June 2019): semi-thin sections for optical microscopy and thin sections for electron microscopy. The semi-thin sections were counterstained with 1% toluidine blue and observed with a Leica DMRB optical microscope equipped with a Leica DC 500 camera (www.leica-microsystems.com, accessed on 14 October 2021). The thin sections were counterstained with uranyl acetate and lead citrate and observed with a JEOL JEM-1011 transmission electron microscope (JEOL, Tokyo, Japan), equipped with a GATAN Orius digital camera (GATAN, Pleasanton, CA, USA) belonging to the microscopy service of the University of Murcia (Spain). 

**Figure 1:**
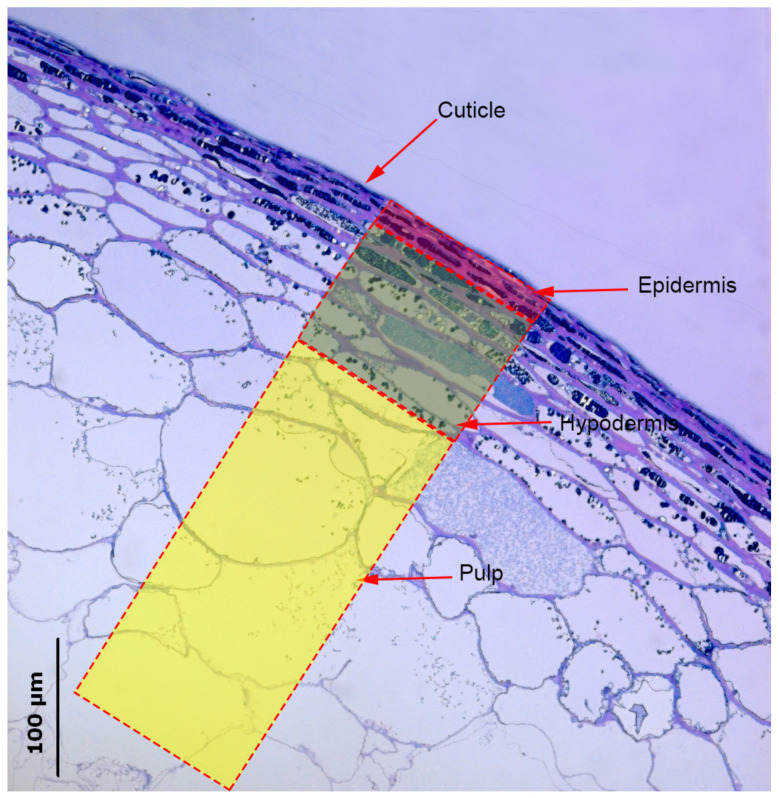
Transverse section of Monastrell berry skin at maturity, taken with a Leica DMRB optical microscope equipped with a Leica DC 500 camera. The cuticle, epidermis, hypodermis, and pulp are indicated.

Measurements of the histological and skin characteristics were conducted using imaging software (ImageJ 1.46; Wayne Rasband; National Institute of Mental Health, Bethesda, Maryland, USA), considering (×40 magnification) the epidermis and hypodermis in each image (Figure 1).

The thickness of the cuticle, epidermis, and hypodermis was measured (μm). The thickness of the cell wall of epidermis and hypodermis cells was also measured. To determine the cell size parameters, the polar and longitudinal diameters of each cell layer (epidermis, hypodermis, and mesocarp) were measured. The cross-sectional area (μm^2^) of the cells in each layer was estimated assuming the shape of this area to be an ellipse.

Skin thickness was estimated as the distance at which the cell sphericity (the ratio of the polar and longitudinal diameters) of the cells increased by 90% with respect to the minimum sphericity (measured in the first layer of cells after the cuticle). The number of cell layers from this point to the cuticle was also estimated. 

**Figure 2:**
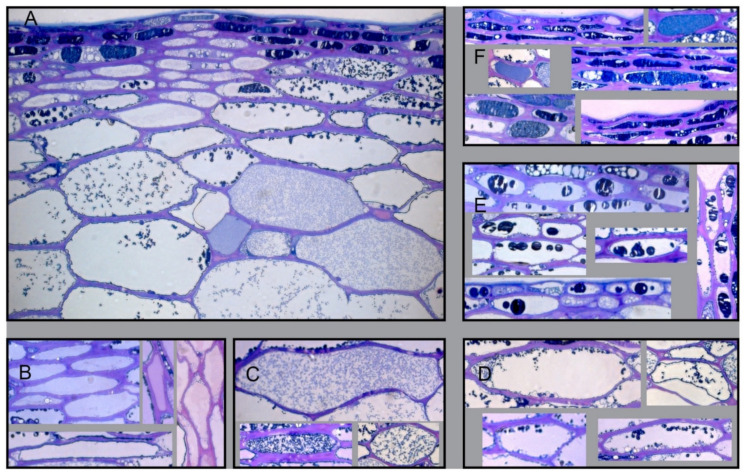
Typology of Monastrell grape skin cells according to the cellular distribution of phenolic compounds found during histological observations (**A**). There were two types of cells: cells without color (without phenolic compounds) and colored cells (varying from uniform coloration to the presence of spherical or shapeless inclusions, either free in the vacuole or adhering to the tonoplast). According to the distribution, size, and shape of the granulations in the vacuole, the skin cells were classified into five classes: cells without any coloration (**B**), cells with fine granulations homogeneously distributed in the vacuole (**C**), cells with small spherical inclusions (**D**), cells with large, round, distorted inclusions (**E**), and cells with uniform coloration (**F**).

According to the cellular distribution of the phenolic compounds (Figure 2), the percentages of each type of cell in the epidermis and hypodermis were quantified. 

### 2.6. Tasting and Sensory Analysis of Grapes

Before the harvest of 2015, samples of 25 mature, healthy, and representative berries were collected randomly from different plots for each R × IS combination and taken to the laboratory. Immediately, a difference tasting was conducted by a consumer panel of five tasters, at room temperature and under normal daylight conditions. The differences between the grapes were assessed through a blind tasting, following the OIV protocols and recommendations. Physical/mechanical/sensorial characteristics of the whole berries, skins, and seeds were observed, valued, and annotated on a tasting sheet. A full sensory evaluation was performed using 11 descriptors that allowed a general characterization of the berry, skin, and seeds. These included two attributes for general berry appearance, three for pulp, three for skin, and three for seeds. The definition and description of these sensory attributes are summarized in Table S1.

### 2.7. Microvinifications and Quality Attributes of Wine

Grapes from the different rootstocks and irrigation methods were collected at harvests in 2014, 2015, and 2016, and used to perform microvinifications. The details of the microvinifications and the analysis of the wine composition and wine quality index (QI_wine_) after alcoholic fermentation have been described previously [16,28].

### 2.8. Statistical Analysis

The data were analyzed using analysis of variance (ANOVA) procedures and means were separated by Duncan’s multiple range test, using Statgraphics Plus 5.1 software (Statistical Graphics Corporation, Warrenton, VA, USA). A three-way ANOVA procedure was used to discriminate the effects of the rootstock, irrigation method, and year. When there was a significant effect (*p*-value < 0.05), means were separated using Duncan’s multiple range test. Linear and nonlinear regressions were fitted using SigmaPlot 11.0 (Systat, Richmond, CA, USA). Schwarz’s Bayesian criterion index (SBC) was used to find the best fit for nonlinear regression between parameters. A total of nine histological and morphological variables of the berry that showed significant differences among the treatments were used to perform a principal component analysis (PCA). This allowed representation of the original data in a smaller space, obtaining new, uncorrelated variables that could be easily interpreted, and was also used to classify—through cluster analysis—the combinations studied (rootstock × irrigation) into subgroups according to their similarity. Furthermore, this PCA technique allowed the quality variables to be correlated with the histological and morphological descriptors obtained. For these studies, the statistical software IBM SPSS Statistics 25 and Statgraphics Centurion 18 Version 18.1.14 were used.

## 3. Results

### 3.1. Histological Analysis of Berry Skin

Histochemical observations made it possible to identify the different tissues that form the skin in Monastrell grapes (Figure 1). The epidermis, covered with a waxy cuticle, had two–three outermost cell layers of compact cells, while the cell layers immediately below the epidermis, with much larger cells, were considered to be the hypodermis [33]. Histological measurements of the different skin fractions (cuticle, epidermis, and hypodermis, Figure 1) are shown in Table 1. Some parameters—such as the cell wall thickness (whole skin and epidermis), layer thickness (whole skin and hypodermis), or pulp cell size—were not modified by the rootstocks. However, other parameters were highly influenced by the rootstock. For example, the cuticle of grapes from vines on the 140Ru rootstock was significantly thicker than in grapes from the other rootstocks.

The cell size of epidermal cells was the smallest one in grapes from the 110R rootstock and largest in those from 41B, 1103P, and 161-49C. Rootstocks 161-49C and 41B also had the largest cell size in the hypodermis, whereas grapes from 110R had the smallest. When the whole skin (epidermis + hypodermis) was considered, no significant differences in the cell size were observed among the rootstocks (Table 1). No differences among the rootstocks were observed in the size of the pulp cells or in the hypodermis/epidermis or pulp/skin ratios.

The analysis also revealed significant effects of the irrigation strategy (PRI—partial root irrigation—vs. RDI—regulated deficit irrigation) and its interaction with the rootstock on the berry skin characteristics. Compared to RDI, PRI significantly increased the cuticle thickness (in 41B and 161-49C), epidermal cell size (140Ru), and epidermal cell size/cell wall thickness ratio (140Ru). In contrast, PRI decreased the wall thickness of the hypodermal cells and the whole skin cells (all rootstocks), the cell size of the whole skin (161-49C), and the epidermal cell size/cell wall thickness ratio (161-49C), compared to RDI. 

Phenolic compounds were mainly located in the vacuoles of grape skin cells (Figure 1), with a positive concentration gradient from the outside to the inside of the grapes. Histochemical observations made it possible to differentiate two types of cells in berry skins: cells without phenolic compounds (absence of coloration) and cells with phenolic compounds (colored cells) [34,35]. The colored cells were differentiated into the following cell types: with uniform coloration, with homogeneously distributed fine granules, with small spherical inclusions (bound to the tonoplast or free in the vacuole), and with large inclusions (Figure 2, Table 2) [36]. A pattern was observed in the location of the different kinds of cells: in the epidermis, cells with uniform coloration were the most abundant; however, in the hypodermis, cells without coloration and cells with small spherical inclusions bound to the tonoplast were predominant (Table 2). The rootstock had little effect on the distribution of these types of cells in the epidermis and hypodermis. Grapes from the 110R rootstock had the highest percentages of cells with a uniform coloration in the epidermis and hypodermis, but also the lowest percentage of cells with large inclusions in the epidermis (Table 2). The irrigation strategy effect on the distribution of these types of cells depended on the rootstock. In the epidermis, PRI increased the percentage of cells without coloration and with large inclusions (140Ru) and decreased the percentage of cells with a uniform coloration (140Ru) and large inclusions (1103P). In the hypodermis, PRI increased the percentage of cells with a uniform coloration (110R) and decreased the percentage of cells with fine granulation (41B).

**Figure 3:**
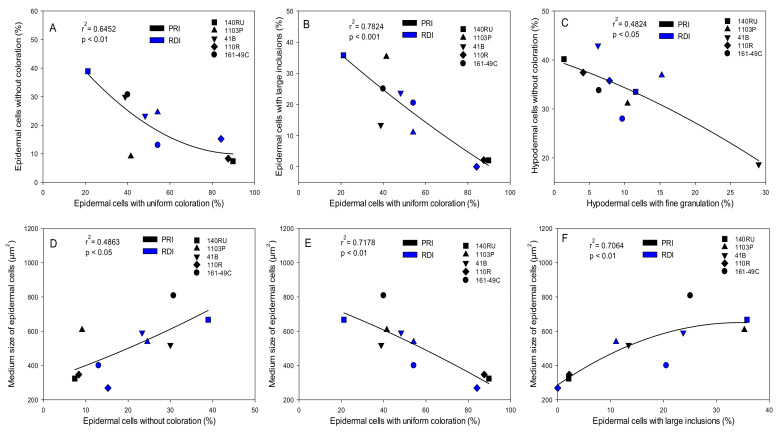
Significant relationships between the percentages of different types of cells in the epidermis and hypodermis of the berry and the morphological characteristics of these cells. Relationships between the percentages of epidermal cells with uniform coloration and epidermal cells without coloration (**A**) and epidermal cells with large inclusions (**B**), percentages of hypodermal cells with fine granulations and cells without coloration (**C**), percentage of epidermal cells without coloration and medium size of epidermal cells (**D**), percentage of epidermal cells with uniform coloration and medium size of epidermal cells (**E**), and percentage of epidermal cells with large inclusions and medium size of epidermal cells (**F**) The data are from 2015.

Some anatomical characteristics of the skin cells were correlated with the distribution of different types of cells in the epidermis and hypodermis. The percentage of cells with a uniform coloration was inversely correlated with the percentages of cells without coloration in the epidermis (r^2^ = 0.6452) and of cells with large inclusions (r^2^ = 0.7824) (Figure 3A,B). In the hypodermis, the percentage of cells without coloration was inversely correlated with the percentage of cells with a fine granulation (r^2^ = 0.4824) (Figure 3C). In the epidermis, the mean cell size was correlated positively with the percentages of cells without coloration and cells with large inclusions (r^2^ = 0.4863 and r^2^ = 0.7064, respectively) (Figure 3D,F), but inversely with the percentage of cells with a uniform coloration (r^2^ = 0.7178) (Figure 3E).

### 3.2. Grape Morphological Characteristics and Texture Analysis

The morphology of the berries of the Monastrell vines was influenced by both the rootstock and the irrigation strategy (Table 3). Although no differences were found for the water content or the pulp or skin weight per berry, the rootstock modified the number, weight, and size of the seeds and the skin/pulp and skin/seed ratios. Hence, rootstocks 110R, 140Ru, and 161-49C had lower seed weights per berry than 41B. Moreover, while 110R had a lower seed number and a smaller seed size, 140Ru and 161-49C had fewer seeds per berry, but the biggest seeds (Table 3). The opposite occurred with 41B and 1103P; their berries had the highest seed weight per berry due to the high number of seeds, although they were the smallest ones (Table 3). The skin/pulp and skin/seed ratios, the relative percentage of skin (its contribution to the total berry weight), and the must percentage were also higher in 110R and 140Ru compared to 1103P or 41B.

The irrigation method modified the number of seeds and their size for the 140Ru rootstock (Table 3). In this case, PRI produced berries with a higher number of seeds than RDI, but they were smaller. However, in 110R berries, PRI produced a significantly lower number of seeds than RDI, but with a similar weight and size. Moreover, PRI significantly decreased the relative percentage of skin and the skin/pulp ratio compared to RDI, mainly in 140Ru and 161-49C (Table 3).

**Figure 4:**
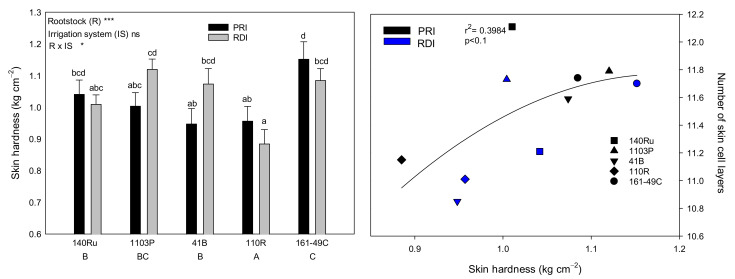
Skin hardness and the relationship between the skin hardness and the number of skin cell layers in Monastrell grapes, for five different rootstocks (140Ru, 1103P, 41B, 110R, and 161-49C) and two different irrigation strategies (PRI and RDI). ns, not significant; *, *p* < 0.05; ***, *p* < 0.001. Bars with different lowercase letters and rootstocks with different capital letters, indicate significant differences according to Duncan’s multiple range test at the 95% confidence level. The data are from 2015.

Significant differences were found in the skin hardness (the maximum force needed to break the skin) of Monastrell berries among the rootstocks; it was lowest for berries from 110R and highest for berries from 161-49C. Moreover, a positive correlation was found between the skin hardness and the number of skin cells (Figure 4).

### 3.3. Total Phenolic Compounds and Total Anthocyanins in Seeds, Skin, and Whole Berries

Total phenolic compounds and one particular type of these compounds, the total anthocyanins, were measured in the skins and seeds and were expressed in different ways (per whole berry and per unit weight of skin, must, and berry) in order to study the possible concentration/dilution effects due to changes in the berry size and/or the must percentage produced by the rootstock or the irrigation strategy (Table 4 and Table 5). The total anthocyanins concentration expressed as mg per g of skin was highest in grapes from 110R, 41B, and 140Ru, and was lowest in grapes from 1103P (Table 4). The rootstocks with the highest concentration of total anthocyanins when calculated as mg per berry, or per g of berry, were also 110R and 140Ru, since, in these grapes, the percentage contribution of the skin to the total berry weight was highest (Table 3). Moreover, although 110R and 140Ru were the rootstocks with the highest must percentages, they also had the highest anthocyanin concentrations on an mg per g of must basis (Table 4). On the other hand, 1103P, the rootstock that produced berries with the lowest skin concentration of total anthocyanins, was also the rootstock with the lowest proportion of skin with regard to the total berry weight (Table 3). As a result, its anthocyanin concentrations expressed as mg per berry or per g of berry fresh weight were also the lowest compared to the other rootstocks (Table 4). This rootstock also gave the lowest total anthocyanins concentration on a must weight basis, despite being one of the rootstocks with the lowest must percentage. The irrigation strategy did not produce any differences in the total anthocyanins found in the skin of the berries (Table 4).

In the same way, the rootstocks with the highest amount of total phenolic compounds in the skin when calculated as mg per weight of skin or berry, or as mg per berry or mg per weight of must, were 110R and 140Ru (Table 5). Moreover, these two rootstocks had the highest concentrations in the must of total phenolic compounds originating from the skin, despite also having the highest must percentages (Table 3). The total phenolic compounds from the seeds did not differ significantly among the rootstocks or between the irrigation methods (Table 5). The concentration of total phenolic compounds of the berry (skin + seeds), calculated per gram of must, was not dependent on the rootstock or the irrigation method. However, when the total phenolic compounds were calculated per berry, we found a strong effect of the rootstock, and the effect was similar to that found in the skin, with the highest concentrations in 110R and 140Ru and the lowest in 41B and 1103P. Most of the total phenolic compounds were from the skin, representing more than 80% of the total phenolic compounds found in the berry (Table 5). The total phenolic compounds concentration in the berry (skin + seed) was not affected significantly by the irrigation method (PRI vs. RDI) or its interaction with the rootstock (R × IS) (Table 5). Due to the variations in the climatic conditions during the study period [15], the year had a strong effect on the skin, seed, and berry total phenolic compounds contents, the berry total phenolic compounds content being highest in 2012 and 2016 and lowest in 2013.

Some important relationships were found between the skin and berry total phenolic compounds and total anthocyanins contents and the percentage distribution of cells by typology in the epidermis and hypodermis (Table 6). The percentage distribution of these cells in the hypodermis did not influence the concentration of total phenolic compounds or total anthocyanins in the skins of Monastrell grapes (Table 6). Similarly, the percentage of cells without coloration or with a fine granulation in the epidermis did not affect the skin concentration of total phenolic compounds or total anthocyanins. The skin total phenolic compounds concentration was negatively correlated with the percentage of cells in the epidermis with small spherical inclusions or large inclusions (Table 6). Moreover, the skin total anthocyanins concentration was also negatively correlated with the percentage of small spherical inclusions found in the epidermis. On the other hand, the percentage of cells with a uniform coloration found in the epidermis was positively correlated with the total phenolic compounds concentration of the skin (Table 6). 

### 3.4. Carbohydrate Concentration in Grapes and Grape Sensorial Analysis

The carbohydrate concentration and composition differed among the rootstocks. Thus, grapes from 161-49C, followed by 110R and 140Ru, showed the highest concentration of sugars (glucose, fructose, and total soluble sugars) compared to 41B or 1103P (Table 7). In addition, 161-49C also had the highest content of starch in the berries. The effect of the irrigation strategy on the carbohydrate composition of the grapes depended on the rootstock. Thus, under PRI, grapes from 161-49C had significantly higher levels of glucose, fructose, and total sugars (G+F+S) and starch than under RDI. Conversely, grapes from 41B had a significantly lower content of starch under PRI. In addition, in 2016, the berries had significantly more sugars and less starch than in the other two years (Table 7).

The most important physical, mechanical, and sensorial characteristics of the whole berries, skins, and seeds were evaluated by a panel of tasters (Table S2). Most of the parameters of the grape sensorial analysis were not modified by the rootstock or the irrigation strategy. The panel only found a significantly higher adherence of the pulp in RDI grapes, relative to PRI, for the 161-49C rootstock (Table S2).

Some of these grape sensorial parameters were significantly correlated with certain physical and chemical berry parameters (Table S3). The maceration skin was positively correlated with the must polyphenol content and with the total and extractable anthocyanins and sugar contents. However, the total and extractable anthocyanins concentrations and QI_phenolic_ were negatively correlated with the skin astringency. The pulp sweetness was correlated negatively with the acidity, OD_520_, and IC of the must, and positively with the tone of the must (Table S3). The peeling of the berries was positively correlated with the tartaric acid and amino acids concentrations.

### 3.5. Must Color

The must color was significantly modified by the rootstock (Table 8). Thus, 140Ru (one of the most vigorous rootstocks) and 41B (medium vigor) gave the lowest CI, OD_620_, OD_520_, and OD_420_ (Table 8). Conversely, the low vigor rootstock 161-49C had the highest values of CI and OD_520_. The most vigorous rootstocks (140Ru and 1103P) gave the highest tone values, whereas the lowest value was found in a low vigor rootstock (161-49C) (Table 8). Differences due to the irrigation strategy were also found. Thus, regardless of the rootstock, PRI resulted in significantly lower values of CI, OD_620_, OD_420_, and tone. Significant interactive effects (R × IS) also revealed that PRI reduced CI and OD_620_ in 1103P, while the tone was reduced by PRI in the must from 140Ru, 1103P, and 161-49C (Table 8). In contrast, the 161-49C PRI grape must had a significantly higher OD_520_ and IC than that from 161-49C RDI.

Regarding the chromatic characteristics measured in 2016, the 140Ru and 110R musts showed the lowest a*, b*, C*, and h*, while the 41B must showed the highest values (Table 8). In all of the rootstocks studied, PRI significantly increased the a*, b*, C*, and h* values with regard to those found in RDI.

### 3.6. Skin Characteristics and Grape, Must, and Wine Quality Relationships

Figure 1 shows a berry skin section with the different tissues of the skin. The outermost three cell layers were considered to be the epidermis, while the four to ten cell layers immediately below were considered to be the hypodermis [33]. Immediately below these two layers, there were cells, polygonal in shape, which were considered to be the pulp. 

**Figure 5:**
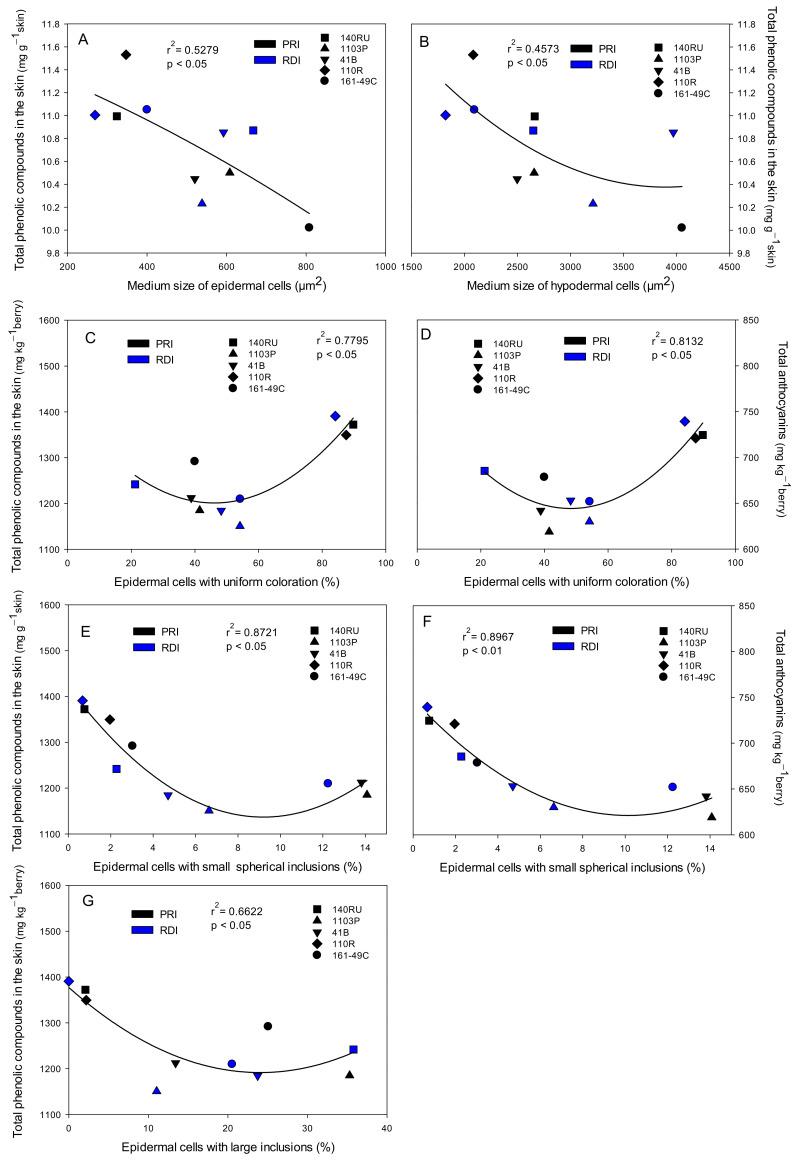
Significant relationships between the skin cell sizes and the percentages of different types of cells in the epidermis and the total phenolic compounds and total anthocyanins concentrations found in the berry skin of Monastrell grapes. Relationships between the total phenolic compounds in the skin and the medium size of epidermal cells (**A**), the medium size of hypodermal cells (**B**), the percentage of epidermal cells with uniform coloration (**C**), the percentage of epidermal cells with small spherical inclusions (**E**), and the percentage of epidermal cells with large inclusions (**G**). Relationships between the total anthocyanins and the percentages of epidermal cells with uniform coloration (**D**) and epidermal cells with small spherical inclusions (**F**). Each point is the average of four years for the total phenolic compounds and total anthocyanins concentrations and one year (2015) for the percentages of each type of cell, calculated per plot for each rootstock.

Some grape and wine quality parameters were correlated with specific skin characteristics. For example, the size of the epidermal and hypodermal cells was negatively correlated with the concentration of total phenolic compounds in the skin (r^2^ = 0.5279 and r^2^ = 0.4573, Figure 5A,B, respectively). Thus, the grapes with the smallest skin cells (hypodermis and epidermis) had the highest concentration of total phenolic compounds per gram of skin. 

Moreover, the skin concentrations of total phenolic compounds and total anthocyanins were correlated positively with the percentage of epidermal cells with uniform coloration (r^2^ = 0.7795 and r^2^ = 0.8132, Figure 5C,D, respectively), but negatively with the percentage of epidermal cells with small spherical inclusions (r^2^ = 0.8721 and r^2^ = 0.8967, Figure 5F,G, respectively). Moreover, the skin concentration of total phenolic compounds was also negatively correlated with the percentage of epidermal cells with large inclusions (r^2^ = 0.6622, Figure 5E).

None of the histological characteristics measured in the skin were correlated with the grape quality parameters such as the polyphenols or anthocyanins (total and extractable) measured in the grape must (Table 9). However, the berry QI_phenolic_ was correlated negatively with the percentage of hypodermal cells without coloration but positively with the percentage of epidermal cells with small spherical inclusions. Moreover, the Seed Maturity index (SM index) was positively correlated with the cuticle thickness and with the percentages of cells with fine granules in the epidermis and hypodermis (Table 9). The wine quality attribute CI was correlated negatively with the size of the epidermal cells, the thickness of the epidermis, and the percentage of cells with small spherical inclusions in the hypodermis but positively with the percentage of hypodermal cells with a uniform coloration (Table 9). The wine anthocyanins concentration was negatively correlated with the skin cell wall thickness, mainly due to the influence of the wall thickness of the hypodermal cells. The global wine quality, measured as the Quality Index (QI_wine_), was negatively influenced by these skin characteristics but it was positively correlated with the percentage of epidermal cells with fine granules (Table 9).

The must color parameters were significantly correlated with the total phenolic compounds in the skin and seeds and with the total anthocyanins concentrations of the Monastrell berries (Table 10). The CI and OD_620_ were positively correlated with the total anthocyanins concentration in the skins when they were expressed per gram of skin, per unit weight, or as a must basis. Additionally, OD_620_, OD_520_, and IC were positively correlated with the total phenolic compounds concentration in the skins and seeds expressed per unit weight of berry or must (Table 10). The total phenolic compounds (skin + seeds) concentration calculated per unit weight of grape was positively correlated with most of the must color parameters (OD_620_, OD_520_, and IC). The total and extractable anthocyanins concentrations found in the must were also strongly correlated with the concentrations of total phenolic compounds and total anthocyanins in the skin, even when they were calculated per unit weight of berry or must (Table 10). Among the parameters related to the maturity of the berry, the Anthocyanins Extractability Index (AE Index) was negatively correlated with the total phenolic compounds and total anthocyanins concentrations found in the berry skins (expressed per unit weight of skin), while the Seed Maturity Index (SM Index) was strongly and negatively correlated with the concentrations of anthocyanins and total phenolic compounds in the skins (Table 10).

The CI of the wine was positively correlated with the total phenolic compounds and total anthocyanins concentrations in the skins, seeds, and berries when they were expressed per unit weight of berry (Table 10). Moreover, the anthocyanins in the wine were correlated directly with the total and extractable anthocyanins and polyphenols contents in the berry, but inversely with the EA and SM indices (Table 11).

### 3.7. Principal Component Analysis of the Berry Characteristics

For the analysis of the main components, nine variables related to different traits of the berries (physical and morphological characteristics of the berry, skin cells typology according to the phenolic compounds distribution, and histological aspects of the skin), which differed significantly among the treatments, were selected. The variables related to the distribution of phenolic compounds in the skins were the most relevant (contributing four variables to the model: percentage of hypodermal cells with a uniform color, and percentages of epidermal cells without color, with a uniform color, and with large inclusions), followed by three histological variables (epidermis thickness and the sizes of the epidermal and hypodermal cells), and, finally, two physical and morphological characteristics of the berry (seed number and the contribution of the seeds to the berry weight). 

**Figure 6:**
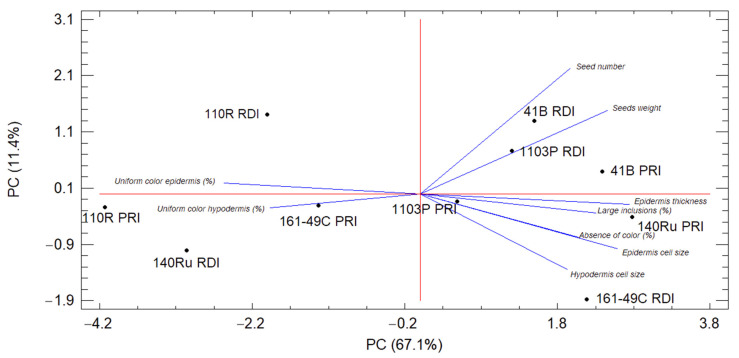
Principal component analysis (PCA) of histological and morphological variables of the berry. Biplot for the first two components (PC). Factorial scores of different combinations (rootstock × irrigation method) and groups determined from cluster analysis, taking into account both components.

A good adaptation of the selected variables was found, with KMO values higher than 0.7 and a highly significant Bartlett’s test. Two components explained 78.5% of the variance, 47.1% being explained by component one (PC1) and 31.3% by component two (PC2). For PC1, large and positive loads were found for five variables (Figure 6): percentage of cells with large inclusions in the epidermis, percentage of cells without coloration in the epidermis, cell size of epidermal cells, cell size of hypodermal cells, and epidermis thickness. Additionally, medium–high loads and negative values were found for two variables: percentage of cells with a uniform coloration in the epidermis and in the hypodermis. For PC2, only two variables with high and positive loads were found, both related to the seed content: seed number and the contribution of the seeds to the berry weight (Figure 6). Thus, PC1 was more related to cellular aspects of the skin, and PC2 to the seeds.

The biplot graphic with the cluster groups of the combinations (Figure 6) was based on their factorial results for PC1 and PC2 and did not show a clear effect of irrigation on the variables. The rootstocks 41B (PRI and RDI) and 1103P (PRI) were grouped in relation to their high seed number (PC2) (Figure 6, Table 3), in contrast to the combinations 110R PRI and 140Ru RDI, with the lowest seed numbers and the highest percentages of cells with a uniform coloration in the epidermis and hypodermis (Table 2). Additionally, 161-49C PRI and 1103P PRI were grouped by their low seed number (Table 3) and their intermediate values for the percentages of cells with a uniform coloration in the epidermis and hypodermis (Table 2). Three combinations were not grouped with any other combination: 110R RDI and 161-49C RDI, with intermediate and high values, respectively, of PC1 and PC2, and 140Ru PRI, with high PC1 and low PC2 values.

**Figure 7:**
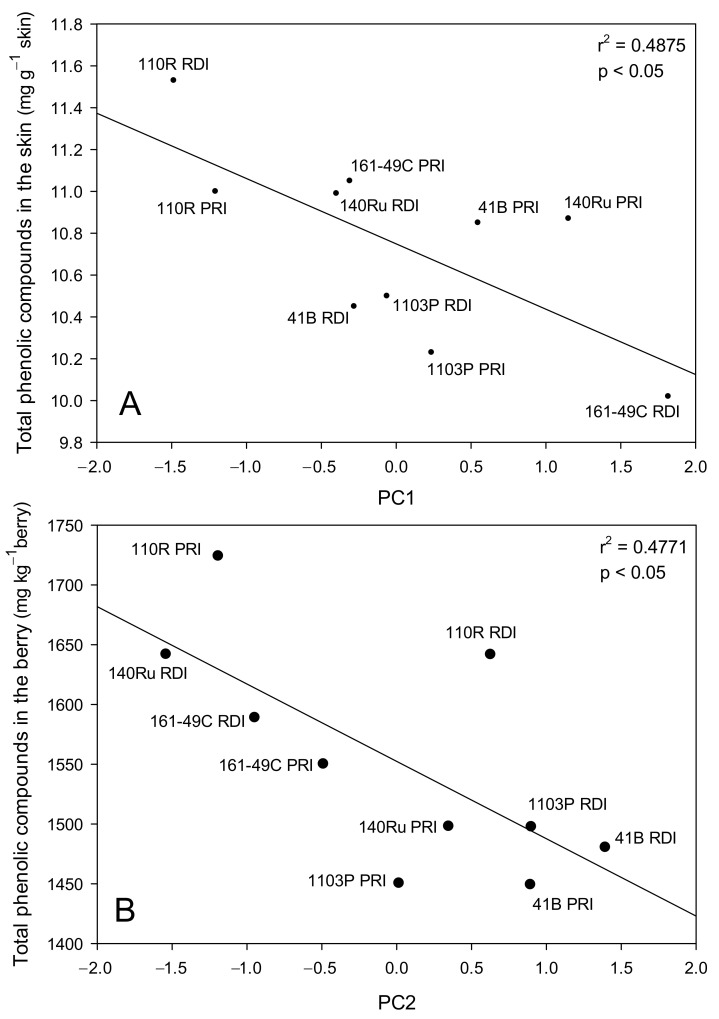
Relationship between histological and morphological descriptors of the berry (PC1 and PC2) and berry quality parameters: (**A**) PC1 versus the content of total phenolic compounds in the skin per g of skin, and (**B**) PC2 versus the content of total phenolic compounds in the berry per kg of berries (**B**). Each point represents the values of these parameters for each combination of rootstock and irrigation strategy.

**Figure 8:**
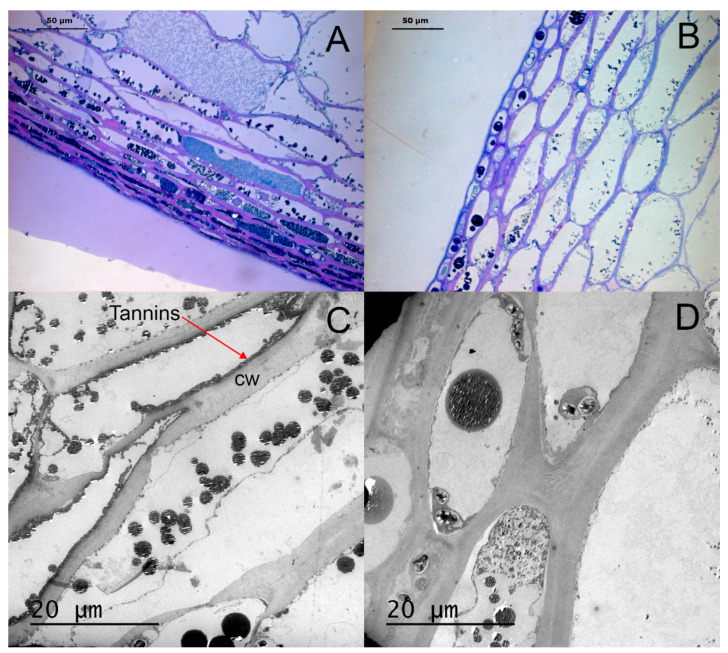
Cross-sections of grape skin of Monastrell grapevines under optical (×40, (**A**,**B**)) and electronic (×1500, (**C**,**D**)) microscopes. The images correspond to grapes from the rootstock 161-49C in the two different irrigation strategies: PRI (**A**,**C**) and RDI (**B**,**D**). (CW, cell wall).

The correlation between the histological and morphological descriptors of the berry obtained by performing PCA with some variables directly related to the berry quality is shown in Figure 7. Significant correlations between PC1 and the content of polyphenols in the skin, on a weight basis, and of PC2 with the total polyphenols content in the berry per unit of berry weight were found. Low total phenolic compounds contents per gram of skin were correlated with high PC1 values (large epidermis and hypodermis cells, high epidermis thickness, and high percentages of cells without phenolic compounds or with large inclusions in the epidermis, Figure 7A). For 161-49C, some important differences were found according to the irrigation regime. Plants of 161-49C receiving RDI (high PC1 values) showed high percentages of both cells without coloration and cells with large inclusions in the epidermis (Table 2), and also had the largest skin cells (Table 1). Optical and electronic microscopy images showed that these grapes had large cells in the epidermis and a high percentage of cells without phenolic compounds or with large inclusions (Figure 8B,D). By contrast, grapes from 161-49C receiving PRI had small cells in both the epidermis and hypodermis (Table 1), and a low percentage of cells without phenolic compounds in the epidermis (Table 2, Figure 8A,C).

## 4. Discussion

### 4.1. Rootstock Effect

The choice of the rootstock had a significant effect on the morphological characteristics of the Monastrell berries; mainly, on the seed characteristics, the skin/pulp/seed proportions of the berry (Table 3), and the accumulation of primary and secondary metabolites in the skin (Table 4 and Table 5) that influenced the final berry and wine quality. The rootstocks that gave the highest final berry quality (161-49C, 110R, 140Ru, in this order [15]) had a series of common morphological characteristics in the berry tissues that were positively related with berry quality, such as: (1) a lower number and weight of seeds per berry, (2) a higher skin/seed ratio, and (3) a higher relative percentage of skin (Table 3).

Moreover, 110R and 140Ru favored a significantly higher concentration of total phenolic compounds and total anthocyanins in the skins (expressed per unit mass of skin) and in the whole berries (expressed per berry) (Table 4 and Table 5). Thus, under deficit irrigation (PRI and RDI), these rootstocks exerted a direct positive effect on the accumulation of total anthocyanins and other phenolic compounds in the berry skins, despite significant differences in berry size, vigor, and yield (e.g., 140Ru vines had 2.6 kg more yield per vine and bigger berries than 110R vines) [15]. In addition, when expressed on a per plant basis, the average annual amount of total skin phenolic compounds and total anthocyanins accumulated in the berries was 7.8 g and 4.3 g per plant, respectively, in 140Ru vines (the highest of all the rootstocks), while in 110R vines, it was 4.8 g and 2.6 g per plant for total skin phenolic compounds and total skin anthocyanins, respectively.

An improvement in the cluster microclimate and increased exposure of the cluster to the sun, due to a lower vine vigor and leaf area development (as observed in low vigor rootstocks such as 110R and 161-49C [15]), and greater water stress during the pre-veraison and ripening periods (as observed in 161-49C [15]), could increase anthocyanin and flavonoids synthesis and accumulation; thus, influencing the compositional changes [17,18,37,38]. The increased accumulation of carbohydrates (glucose, fructose, and starch, Table 7) in the 161-49C berries, mainly under PRI (the most water-stressed plants [15]), indicated the accumulation of metabolites as osmoprotectants and compatible solutes to mitigate the negative effects of stress [15]. However, the most vigorous and productive rootstock (140Ru) had the highest total phenolic compounds content in the skins and berries, indicating the greater potential of this rootstock to promote the accumulation of flavonoids in the berries, regardless of the improvement in the cluster microclimate. 

In contrast, the rootstocks 1103P and 41B had lower concentrations of total phenolic compounds and total anthocyanins in the berry skins, compared to the other rootstocks, suggesting a lower biosynthesis and the accumulation of anthocyanins and other phenolic compounds under deficit irrigation. Moreover, the berries from these rootstocks had the highest number and weight of seeds per berry, the lowest relative percentage of skin and skin/seed ratio (Table 3), and the highest seed maturity index [15]. This indicated a higher tannin content of the seeds and, thus, a greater risk of a negative effect on the flavor of the wine [39], which could explain the greater tannin concentration and astringency reported in these wines and the negative impact on the sensory perception and the final berry and wine quality [15,16]. In addition, according to the PC2 in the analysis of the main components, seed parameters (the number of seeds or their contribution to the total berry weight) were important in the explanation of the variability of the system (Figure 6) and they played an important role in berry quality. In this sense, PC2 was also related to the berry quality, so rootstocks giving a high seed number and seed weight also provided lower contents of total phenolic compounds per berry (Figure 7B). Thus, grapes from the rootstocks 1103P and 41B, with the highest values of seed number and seed weight (Table 3), also had the lowest values of the total phenolic compounds content, a parameter closely related to the final quality of the grapes (Table 5), and produced low-quality wines [16]. Significant differences in the skin and berry phenolic compounds content among rootstocks have been observed previously [13,40]. A higher seed number and higher total seed phenolic compounds in 1103P-grafted vines, compared to other rootstocks, have been reported previously [40]. 

In addition, grape and wine quality could be influenced by other factors different from the phenolic and anthocyanin potential of the grapes. Thus, the smaller berries of low vigor rootstocks could have enhanced the concentration of metabolites in the berries, must, and wines, due to a greater concentration effect. For example, the rootstock 161-49C showed the lowest yield, a low berry weight, a high EA Index [15], and a moderate concentration of total anthocyanins in the skin (lower concentration of total anthocyanins per berry than 110R, Table 4), but it had one of the lowest must percentages (Table 3), which could produce a greater concentration effect. This was reflected in a higher concentration of total and extractable anthocyanins and polyphenols in the must of grapes from 161-49C, compared to other rootstocks [15]. Monastrell grapes from 161-49C also produced wines with significantly more derivatives of anthocyanins and flavonols and, in general, better values of quality parameters related to the phenolic content than other rootstocks such as 110R and 140Ru [16]. Conversely, berries from 110R and 140Ru had greater total phenolic compounds and total anthocyanins accumulation in the skins (Table 4 and Table 5), but bigger berries and higher must percentages compared to other rootstocks (for example, 161-49C) (Table 3). Bigger berries with a higher water content (in the skin and pulp) probably produced a significant dilution effect and reduced the total concentration of flavonoids in the must and wine [28], especially in 140Ru, resulting in lower total and extractable anthocyanins in the must and wine compared to 161-49C [15]. 

Overall, the total phenolic compounds content of the berry (taking into account the skins and seeds, Table 5) was significantly higher in 110R, followed by 140Ru and 161-49C, which was also associated with a better sensory perception and a better final quality of the wines derived from these rootstocks (mainly for 161-49C and 110R [16]). Thus, our study revealed a significant positive correlation between the total phenolic compounds and total anthocyanins concentrations of the grapes and the color parameters and anthocyanins of the must and the color intensity of the wine (Table 10). 

However, grapes with high phenolic compounds concentrations do not always produce musts and wines with high phenolic compounds concentrations; this can be related, among other factors, to the difficulty of the extraction of phenolic compounds from grape skins into musts [39,41]. The high Anthocyanin Extractability Index (AE) found in Monastrell grapes, compared to other varieties such as Cabernet Sauvignon, Merlot, or Syrah, indicates a high rigidity of the cell wall structure in Monastrell grapes, which hinders anthocyanin extraction [19,20,42] since the skin cell wall is a barrier to free phenolic compounds diffusion. In our study, in general, the highest AE Index values of grapes were correlated with the lowest anthocyanins concentrations in wines (Table 11), indicating the difficulty of anthocyanins extraction from the skin to the must during winemaking. Moreover, a higher SM Index (indicating poor berry ripeness and a high contribution of tannins from the seeds [39]) was also significantly related to lower anthocyanins and phenolic compounds contents in the skin cells (Table 10) and wine (Table 11). 

We also identified some changes in specific skin traits that could also have contributed to the differences found among rootstocks in the final berry and wine quality. The skin is the grape’s dermal system and it only represented 11–12% of the fresh weight of Monastrell berries (Table 3). However, the skin is one of the most important issues in wine quality, since anthocyanins are mainly located in the vacuoles of grape skin cells [39] and most of the phenolic compounds of grapes are located therein as cell-wall phenols, confined in the vacuoles or associated with the cell nucleus [43]. Therefore, from the analysis of skin–berry–must–wine relationships (Table 6, Table 9, Table 10 and Table 11, Figure 5), we identified several positive skin features that were related to an increase in total phenolic compounds and total anthocyanins contents and an improvement in final berry and wine quality: (1) lower cuticle and cell wall thickness, (2) lower skin thickness, (3) lower skin (epidermis) cell size, (4) higher percentage of skin cells with a uniform coloration, and (5) lower percentage of cells in the epidermis with small spherical or big colored inclusions.

#### 4.1.1. Cuticle and Cell Wall Thickness

The cuticle serves as a protective barrier against physical, chemical, and biological attack [29] and can minimize water loss and protect against berry cracking [44]. However, as a physical barrier against the internal tissues, a greater thickness could also make it more difficult to extract skin phenolic compounds. Overall, the cuticle thickness of the berries had a small range (2.0–2.5 µm), similar to values found in other varieties (1.5–4.0 µm [39]). Grapes from the 140Ru rootstock had high skin and berry total phenolic contents (Table 5), but also the thickest cuticle compared to other rootstocks (Table 1); this could have hindered the extraction of phenolic compounds from the skin into the must and contributed to the lower total phenolic compounds concentration in the must of grapes from 140Ru [15]. Additionally, the cell wall is a stronger barrier for the phenolic compounds located inside the skin cells. The larger quantities of skin cell wall material in Monastrell skins, compared to other varieties [20], may partly explain the difficulty in extracting phenolic compounds from Monastrell grapes during winemaking. We found significant negative correlations of the cell wall thickness (mainly for hypodermal cells, Table 9) with the wine concentration of anthocyanins and the index QI_wine_; these support the importance of the physical characteristics of the cell wall in the anthocyanin extractability.

#### 4.1.2. Skin Thickness and Skin Cell Size 

The skin thickness is related to the anthocyanin extractability: thinner skins seem to be characterized by higher anthocyanin extractability [45]. In our study, a greater thickness of the epidermis coincided with a lower value of the color intensity in the wine (Table 9). Berries from the 110R rootstock had thinner skins (mainly the epidermis) and thinner cell walls (in the hypodermis) compared to other rootstocks (Table 1), suggesting better/easier anthocyanins/phenolic compounds extraction. This could explain the high concentrations of total phenolic compounds and total anthocyanins in the 110R musts and wines compared to other rootstocks such as 41B and 1103P [15,16]. The thinner skins in 110R were due not to a lower number of skin cells, but to the smaller epidermal and hypodermal cells (Table 1). The high correlation between the size of the epidermal and hypodermal cells and the total phenolic compounds concentration in the skins of Monastrell grapes, expressed on a skin weight basis (Figure 5A,B), suggests that smaller skin cells (higher area/volume ratios) have higher total phenolic compounds concentrations. Thus, rootstocks such as 110R, which produce grapes with small skin cells, have higher total phenolic compounds concentrations in their skin and, therefore, a higher phenolic potential. On the other hand, smaller skin cells mean tighter packing of cells that could produce greater resistance to skin cell breakage. However, skin hardness was not correlated with the cell size, but was correlated with the number of skin cell layers (Figure 4), 110R grapes having the lowest skin hardness. Additionally, along with the cell size, the cell wall thickness must be taken into account since a lower cell size/cell wall thickness ratio could mean thicker walls and a stronger barrier for the phenolic compounds located inside. However, the lowest cell size/cell wall thickness ratio was found in grapes from 110R, which also had the lowest skin hardness values; therefore, other factors, such as the cell wall composition, could be important. High levels of galacturonic acid, mannose, apiose, and glucuronic acid would enhance the extraction of anthocyanins, while glucose, rhamnose, 2-O-methylxylose, and lignin would prevent anthocyanins extraction from skins [46]. Therefore, the cell wall composition is also an important factor to take into account when anthocyanins extractability is studied [20,46].

#### 4.1.3. Typology of Cells in the Skin 

The phenolic berry quality was correlated with the cell typology in the epidermis and hypodermis (Figure 5 and Figure 7, Table 6 and Table 9). The highest percentages of cells with a uniform coloration in the epidermis coincided with the highest total phenolic compounds and total anthocyanins concentrations in the skin (Figure 5C,D). Some authors have found a relationship of the percentage of colored cells with grape maturation [27] or with the mesoclimatic conditions [36]. We found that the rootstock also modified the distribution of the different types of cells in the epidermis and hypodermis, the grapes from 110R having the highest percentages of cells with a uniform coloration (in the epidermis and hypodermis, Table 2), traits that were related positively to the phenolic compounds concentration in the skin and berry (Figure 5 and Figure 7, Table 6 and Table 9). It has been suggested that, in the variety Tempranillo, a higher concentration of phenolic compounds during winemaking was related to higher percentages of cells with larger inclusions in the epidermis and the first layers of the hypodermis [27]. However, no correlation between the percentage of cells with large inclusions in the skin of Monastrell grapes and the berry quality appeared in our study, but there was a correlation between the percentage of skin cells with a uniform coloration and the total phenolic compounds and total anthocyanins in the skin of Monastrell berries (Figure 5C,D). According to these relationships, it seems that percentages of up to 50% of cells with a uniform coloration increased the concentrations of total phenolic compounds and total anthocyanins in the skin. In the same way, a positive correlation was obtained between IC and QI_wine_ and the percentages of hypodermal cells with a uniform coloration and of epidermal cells with fine granules, respectively (Table 9). 

In addition, the analysis of the main components revealed the importance of the distribution of phenolic compounds in the skin cells with regard to explaining the variability of the berry system (Figure 6). In the epidermis, variables such as the percentage of cells with uniform coloration, with large inclusions, or without phenolic compounds, as well as the thickness and cell size, were especially relevant. Moreover, the significant correlation of PC1 with the content of phenolic compounds in the skin indicated that some physical and morphological parameters of the grapes are important to the final quality of the grape (Figure 7). Low phenolic compounds contents (161-49C RDI), which would be associated with a worse grape quality, were correlated with high percentages of cells without phenolic compounds or with large inclusions in the epidermis, and also with large skin cells and a high epidermal thickness (Figure 7A). 

### 4.2. Effects of the Irrigation Method and Its Interaction with the Rootstock

Compared to RDI, PRI did not significantly alter the contents and concentrations of total phenolic compounds and total anthocyanins in the skins, seeds, and berries of Monastrell vines (Table 4 and Table 5). In contrast, PRI significantly modified some specific morphological/anatomical skin/berry traits, resulting in a higher cuticle thickness, lower cell wall thickness (mainly in the hypodermis), a lower percentage of skin, and a lower skin/pulp ratio. These may have contributed to important changes in the final concentration of phenolic compounds in the musts and wines, depending on the rootstock. In fact, some significant interactions between the rootstock and irrigation method were observed for different skin/berry characteristics (Table 1, Table 2 and Table 3). In low vigor vines (161-49C and 110R), some common features, highly correlated with the grape and wine quality (Table 9), were modified by PRI, when compared to RDI. For example, smaller cells in the epidermis and hypodermis of the skin (161-49C), a higher percentage of cells with a uniform coloration in the hypodermis (110R), or a lower number of seeds per berry (161-49C) may have contributed to the better phenolic quality of the must and wines observed for these rootstocks under PRI [15,16]. In this sense, the combination that produced the lowest values of the wine quality indicators [16], 161-49C with RDI, showed high percentages of cells without phenolic compounds or with large inclusions of phenolic compounds and also had large skin cells (Table 1 and Table 2, Figure 8). However, when these vines received PRI, the grapes had smaller skin cells and a lower percentage of cells without phenolic compounds in the epidermis (Table 1 and Table 2, Figure 8), significantly improving the must and wine quality (with the highest must QI_phenolic_ and QI_wine_ [15,16]).

In contrast, under PRI, berries from 41B or 140Ru (medium and high-vigor vines) showed a significantly greater epidermis thickness (140Ru), higher cuticle thickness (41B), higher number of seeds (140Ru), lower skin/pulp ratio and percentage of skin (140Ru), greater percentage of cells in the epidermis without coloration or with large inclusions, and lower percentage of cells with a uniform coloration in the epidermis (140Ru). These may have negatively impacted the final phenolic compounds concentration in the must of PRI grapes from these rootstocks [15,16].

## 5. Conclusions

The rootstock had a great influence on the anatomical and morphological characteristics of Monastrell grapes and on the accumulation of phenolic compounds in the skin and seeds. Rootstocks of different vigor, such as 110R and 140Ru, accumulated great amounts of total phenolic compounds and total anthocyanins in the skin, which is important to achieve a high final wine quality. However, although the phenolic concentration in the skin is important, other factors also have a great impact on the phenolic compounds concentration in the must. Despite having high concentrations of total phenolic compounds and total anthocyanins in the skin, 110R and 140Ru also had the biggest grapes and the highest must percentages, which lowered the concentrations of metabolites in the must and wine. Berries from the rootstock 161-49C, with moderate concentrations of total phenolic compounds and total anthocyanins in the skin, had a low berry weight, a low must percentage, and a low pulp/skin cell size ratio, factors that contribute to an increase in the concentration of phenolic compounds in the must.

Some histological differences found in the grapes from different rootstocks could also have contributed to the differences seen in the final concentration of phenolic compounds in the must. The most relevant layer in this study was the epidermis. Small epidermal cells led to the accumulation of high amounts of phenolic compounds and total anthocyanins in the skin, increasing the skin phenolic compounds concentration and the must and wine quality. Grapes from 110R had the smallest cells in both the epidermis and hypodermis, and the highest concentration of these compounds in the skin. Moreover, the percentage of cells with a uniform coloration influenced the total phenolic compounds and total anthocyanins in the skin of Monastrell grapes, which were also related to the final quality of the wine.

In summary, the final quality of the grape—and, thus, the must and wine—is closely related to histological and morphological aspects, and significant changes in these aspects, specifically in the skin layer, can be produced according to the choice of rootstock and irrigation strategy. In the context of adaptation to global warming, in order to increase the phenolic quality of grapes and wines in different varieties and edaphoclimatic conditions, more research is needed on rootstock effects and deficit irrigation strategies on anatomical/morphological/metabolomic changes of different berry compartments (such as skins and seeds), to select the most suitable rootstocks and optimize the irrigation strategies.

## Figures and Tables

**Table 1:** Anatomical/histological characteristics of the cuticle, cell wall, skin, and pulp cells of Monastrell grapes, on five different rootstocks (140Ru, 1103P, 41B, 110R, and 161-49C) and for two different irrigation strategies (PRI and RDI), in 2015.

Rootstock (R)		Cuticle Thickness (µm)	Cell Wall Thickness (µm)			Thickness (µm)			Cell Size (µm^2^)						Epidermis Cell Size/Cell Wall Thickness
			Whole Skin	Epidermis	Hypodermis	Whole Skin	Epidermis	Hypodermis	Whole Skin	Epidermis	Hypodermis	Hypodermis/Epidermis	Pulp	Pulp/Skin	
140Ru		2.50 b	2.80	2.08	3.11 ab	156	17.7 b	138	2008	496 ab	2656 ab	6.2	17,064	9.9	237 b
1103P		2.31 a	2.87	2.16	3.18 ab	163	17.9 b	145	2226	573 b	2935 ab	5.3	24,479	11.7	271 b
41B		2.18 a	2.69	2.33	2.85 a	162	19.6 b	142	2431	556 b	3234 b	5.8	25,193	10.9	256 b
110R		2.13 a	2.78	2.14	3.06 a	134	13.0 a	121	1459	308 a	1952 a	6.9	22,462	16.5	150 a
161-49C		2.27 a	3.07	2.24	3.44 b	195	18.4 b	177	2283	604 b	3073 b	5.1	17,606	11.3	245 b
**Irrigation strategy (IS)**															
PRI		2.39	2.73	2.12	2.99	165	17.4	147	2053	493	2750	5.8	20,199	11.6	240
RDI		2.17	2.96	2.26	3.27	159	17.2	142	2110	521	2790	5.9	22,524	12.6	223
**R** **× IS**															
140Ru	PRI	2.50 cd	2.67	2.05	2.93	159	21.0 c	138	2054 ab	667 bc	2649 ab	4.3	12,590	7.1	335 c
	RDI	2.50 cd	2.93	2.10	3.29	152	14.5 ab	138	1961 ab	324 ab	2663 ab	8.1	21,538	12.7	140 a
1103P	PRI	2.39 bcd	2.70	2.24	2.90	187	17.0 bc	170	2410 ab	538 abc	3212 ab	6.0	21,836	9.9	254 abc
	RDI	2.23 abc	3.05	2.08	3.46	139	18.8 c	121	2042 ab	608 abc	2657 ab	4.5	27,123	13.5	288 bc
41B	PRI	2.35 bcd	2.78	2.15	3.06	189	20.4 c	168	2957 b	592 abc	3970 b	6.8	25,912	9.0	288 bc
	RDI	2.01 a	2.60	2.51	2.64	135	18.7 bc	116	1905 ab	520 abc	2498 ab	4.8	24,475	12.9	225 abc
110R	PRI	2.12 a	2.59	1.93	2.88	124	11.6 a	112	1356 a	269 a	1822 a	6.8	21,343	16.0	149 a
	RDI	2.14 ab	2.98	2.34	3.25	145	14.5 ab	131	1562 a	347 ab	2083 a	7.1	23,581	17.0	150 a
161-49C	PRI	2.60 d	2.88	2.23	3.19	167	17.1 bc	150	1488 a	400 ab	2094 a	5.3	19,312	15.8	177 ab
	RDI	1.95 a	3.25	2.24	3.70	224	19.8 c	204	3078 b	808 c	4051 b	4.9	15,900	6.8	314 c
**ANOVA**															
R		***	ns	ns	*	ns	**	ns	ns	*^⊥^	*^⊥^	ns	ns	ns	*
IS		***	*	ns	*	ns	ns	ns	ns	ns	ns	ns	ns	ns	ns
R × IS		**	ns	ns	ns	ns	*	ns	*	*	*	ns	ns	ns	**

ns, not significant; *, **, and *** indicate significant differences at the 0.05, 0.01, and 0.001 levels of probability, respectively. In each column and for each factor or interaction, different letters indicate significant differences according to Duncan’s multiple range test at the 95% confidence level. **^⊥^** In the epidermis and hypodermis, cell size differences among rootstocks were significant at the 0.07 level of probability.

**Table 2:** Percentage distribution of cells by typology in the epidermis and hypodermis of Monastrell grape skins, for five different rootstocks (140Ru, 1103P, 41B, 110R, and 161-49C) and two different irrigation strategies (PRI and RDI), in 2015. Types of cell: absence of coloration and with coloration (with uniform coloration, with homogeneously distributed fine granules, with small spherical inclusions bound to the tonoplast or free in the vacuole, and with large inclusions).

Rootstock (R)		Epidermis (1–3 Layers)					Hypodermis (4–10 Layers)				
		Absence of Coloration (%)	Uniform Coloration (%)	Fine Granulation (%)	Small Spherical Inclusions (%)	Large Inclusions (%)	Absence of Coloration (%)	Uniform Coloration (%)	Fine Granulation (%)	Small Spherical Inclusions (%)	Large Inclusions (%)
140Ru		23.2	55.5 a	0.9	1.5	18.9 b	39.8	3.7 a	7.0	41.0	8.5
1103P		16.8	47.8 a	1.8	10.4	23.26 b	34.4	4.0 a	13.0	42.6	6.1
41B		26.6	43.5 a	2.0	9.3	18.6 b	34.9	2.1 a	18.7	40.5	3.8
110R		11.8	85.8 b	0.0	1.3	1.1 a	37.0	13.3 b	6.0	37.0	6.7
161-49C		21.9	47.1 a	0.6	7.6	22.8 b	32.9	7.9 ab	8.5	44.2	6.5
**Irrigation strategy (IS)**											
PRI		23.0	52.4	1.1	5.3	18.2	37.8	7.8	10.6	39.5	4.3
RDI		17.1	59.5	1.0	6.7	15.6	33.8	4.6	10.6	42.6	8.4
**R** **× IS**											
140Ru	PRI	38.9 c	21.2 a	1.8	2.3	35.8 c	36.1	2.4 a	12.4 ab	45.5	3.5
	RDI	7.4 a	89.8 c	0.0	0.8	2.1 ab	43.6	5.0 a	1.5 a	36.5	13.4
1103P	PRI	24.5 abc	54.2 abc	3.6	6.6	11.0 ab	37.2	0.8 a	15.4 bc	43.2	3.4
	RDI	9.1 ab	41.5 a	0.0	14.1	35.3 c	31.6	7.2 a	10.6 ab	42.0	8.7
41B	PRI	23.3 abc	48.3 ab	0.0	4.7	23.7 abc	50.4	1.0 a	7.3 ab	39.7	1.6
	RDI	30.0 abc	38.8 a	4.0	13.8	13.4 abc	19.4	3.2 a	30.1 c	41.2	6.0
110R	PRI	15.2 ab	84.1 bc	0.0	0.7	0.0 a	35.8	22.5 b	7.9 ab	29.2	4.7
	RDI	8.3 ab	87.5 c	0.0	2.0	2.2 ab	38.2	4.1 a	4.2 ab	44.8	8.7
161-49C	PRI	13.0 ab	54.2 abc	0.0	12.3	20.5 abc	29.6	12.3 a	10.3 ab	39.8	8.0
	RDI	30.7 bc	40.0 a	1.2	3.0	25.1 bc	36.2	3.5 a	6.8 ab	48.6	5.0
**ANOVA**											
R		ns	**	ns	ns	*	ns	*	ns	ns	ns
IS		ns	ns	ns	ns	ns	ns	ns	ns	ns	ns
R × IS		**	**	ns	ns	**	ns	*	*	ns	ns

ns, not significant; * and ** indicate significant differences at the 0.05 and 0.01 levels of probability, respectively. In each column and for each factor or interaction, different letters indicate significant differences according to Duncan’s multiple range test at the 95% confidence level.

**Table 3:** Morphological parameters of the Monastrell berries, for five different rootstocks (140Ru, 1103P, 41B, 110R, and 161-49C) and two different irrigation strategies (PRI and RDI), from 2012 to 2016.

Rootstock (R)		H_2_O (%)	mg Pulp Berry^−1^	mg Skin Berry^−1^	mg Seed Berry^−1^	Seed Number	mg Seed Seed^−1^	Skin/Pulp Ratio	Skin/Seeds Ratio	Skin (%)	Pulp (%)	Seed (%)	Must (%)
140Ru		71.0	1370	185	80.6 a	2.04 a	40.1 c	0.149 b	2.27 c	11.9 bc	83.0	5.2	63.3 b
1103P		70.7	1321	176	83.7 ab	2.26 bc	37.4 ab	0.137 a	2.16 ab	11.2 a	83.4	5.4	60.9 a
41B		71.1	1343	177	87.5 b	2.37 c	37.0 a	0.139 a	2.14 a	11.3 ab	83.7	5.6	60.2 a
110R		70.1	1294	181	78.9 a	2.12 ab	37.3 ab	0.149 b	2.26 c	12.1 c	82.6	5.3	64.1 b
161-49C		70.7	1298	181	80.4 a	2.09 a	38.8 bc	0.143 ab	2.23 bc	11.9 abc	82.9	5.3	60.9 a
**Irrigation strategy (IS)**													
PRI		70.7	1338	178	81.9	2.17	38.0	0.139	2.19	11.4	83.3	5.3	62.0
RDI		70.7	1313	182	82.6	2.18	38.2	0.148	2.24	12.0	82.9	5.4	61.8
**Year**													
2012		-	1319 b	184 b	82.0 b	2.11 ab	39.2 bc	0.140	2.29	11.6 c	83.2 b	5.2 b	68.4 b
2013		-	1636 c	202 c	93.5 c	2.40 c	39.4 c	0.124	2.21	10.5 b	84.6 c	4.9 ab	58.9 a
2014		-	1335 b	146 a	71.9 a	2.02 a	36.0 a	0.110	2.06	9.5 a	85.9 d	4.7 a	-
2016		70.7	1012 a	188 b	81.4 b	2.18 b	37.9 b	0.195	2.33	15.1 d	78.9 a	6.5 c	58.4 a
**R** **× IS**													
140Ru	PRI	70.6	1421	186	84.8	2.22 cde	38.6 b	0.139 a	2.18	11.3 a	83.5	5.2	62.3
	RDI	71.4	1318	183	76.3	1.86 a	41.5 c	0.159 c	2.36	12.4 bc	82.5	5.1	64.3
1103P	PRI	70.8	1309	173	82.3	2.21 cde	37.4 ab	0.137 a	2.16	11.1 a	83.6	5.3	61.0
	RDI	70.5	1333	180	85.2	2.31 de	37.3 ab	0.137 a	2.16	11.3 a	83.3	5.4	60.9
41B	PRI	71.5	1355	173	87.2	2.37 e	37.1 ab	0.134 a	2.07	10.9 a	83.6	5.5	60.4
	RDI	70.8	1332	181	87.8	2.37 e	37.0 ab	0.144 ab	2.21	11.7 ab	83.9	5.6	60.0
110R	PRI	70.8	1276	183	76.4	1.99 ab	38.6 ab	0.157 bc	2.35	12.5 bc	82.2	5.3	64.7
	RDI	69.3	1312	180	81.3	2.26 cde	36.1 a	0.141 abc	2.16	11.7 ab	83.0	5.3	63.4
161-49C	PRI	69.9	1328	173	78.7	2.07 bc	38.3 ab	0.128 a	2.17	11.0 a	83.9	5.1	61.5
	RDI	71.4	1269	188	82.2	2.10 bcd	39.3 b	0.159 c	2.29	12.7 c	81.8	5.5	60.3
**ANOVA**													
R		ns	ns	ns	*	***	***	*	**	*	ns	ns	***
IS		ns	ns	ns	ns	ns	ns	**	ns	**	ns	ns	ns
Year		-	***	***	***	***	***	***	**	***	***	***	***
R × IS		ns	ns	ns	ns	***	*	**	ns	**	ns	ns	ns

ns, not significant; *, **, and *** indicate significant differences at the 0.05, 0.01, and 0.001 levels of probability, respectively. In each column and for each factor or interaction, different letters indicate significant differences according to Duncan’s multiple range test at the 95% confidence level.

**Table 4:** The concentration of total anthocyanins in Monastrell grapes, for five different rootstocks (140Ru, 1103P, 41B, 110R, and 161-49C) and two different irrigation strategies (PRI and RDI), from 2014 to 2016. The concentrations are expressed in different units: mg g^−1^ skin, mg berry^−1^, mg g^−1^ berry, and mg g^−1^ must.

	Total Anthocyanins			
Rootstock (R)	mg g^−1^ Skin	mg Berry^−1^	mg g^−1^ Berry	mg g^−1^ Must
140Ru	6.00 b	1.08 bc	0.71 bc	1.21 bc
1103P	5.32 a	0.93 a	0.60 a	1.01 a
41B	5.84 b	0.99 ab	0.65 ab	1.10 ab
110R	6.09 b	1.09 c	0.73 c	1.24 c
161-49C	5.67 ab	1.00 ab	0.67 b	1.13 abc
**Irrigation strategy (IS)**				
PRI	5.83	1.01	0.66	1.13
RDI	5.74	1.03	0.68	1.14
**Year**				
2012	7.19 c	1.30 c	0.83 c	1.21 b
2013	4.25 a	0.86 a	0.44	0.74 a
2014	5.96 b	0.86 a	0.56 b	-
2016	5.74 b	1.05 b	0.85 c	1.45 c
**ANOVA**				
Rootstock (R)	*	**	***	**
Irrigation strategy (IS)	ns	ns	ns	ns
Year	***	***	***	***
Interaction (R × IS)	ns	ns	ns	ns

ns, not significant; *, **, and *** indicate significant differences at the 0.05, 0.01, and 0.001 levels of probability, respectively. In each column and for each factor or interaction, different letters indicate significant differences according to Duncan’s multiple range test at the 95% confidence level.

**Table 5:** The skin, seeds, and total grape phenolic contents and the contributions of the skin and seeds to the total phenolic content of Monastrell grapes, for five different rootstocks (140Ru, 1103P, 41B, 110R, and 161-49C) and two different irrigation strategies (PRI and RDI), from 2014 to 2016. The concentrations are expressed in different units: mg g^−1^ skin, mg g^−1^ berry, mg g^−1^ must, and mg berry^−1^.

	Total Phenolic Compounds Content											
	Skin				Seeds				Berry		Contribution	
Rootstock (R)	mg g^−1^ Skin	mg Berry^−1^	mg g^−1^ Berry	mg g^−1^ Must	mg g^−1^ Seed	mg Berry^−1^	mg g^−1^ Berry	mg g^−1^ Must	mg Berry^−1^	mg g^−1^ Must	% Skin	% Seed
140Ru	10.9 ab	1.98 b	1.31 bc	2.28 bc	4.8	0.38	0.26	0.45	2.37 bc	2718	83.5	16.5
1103P	10.4 a	1.80 a	1.17 a	2.02 a	5.6	0.45	0.31	0.59	2.26 ab	2589	80.0	20.0
41B	10.7 a	1.82 a	1.20 a	2.08 ab	4.6	0.39	0.27	0.51	2.19 a	2476	83.0	17.0
110R	11.3 b	2.00 b	1.37 c	2.32 c	5.7	0.43	0.31	0.55	2.45 c	2827	82.8	17.2
161-49C	10.5 a	1.86 a	1.25 ab	2.16 abc	6.0	0.46	0.32	0.58	2.31 ab	2713	80.4	19.6
**Irrigation strategy (IS)**												
PRI	10.8	1.88	1.24	2.14	5.6	0.44	0.30	0.53	2.31	2621	81.3	18.7
RDI	10.7	1.91	1.28	2.21	5.2	0.41	0.29	0.54	2.32	2708	82.6	17.4
**Year**												
2012	13.0 d	2.36	1.50 c	2.20 b	3.9 ab	0.31	0.20 a	0.29 a	2.67 b	2490 b	88.3 c	11.7 a
2013	7.5 a	1.50	0.78 a	1.32 a	3.7 a	0.35	0.18 a	0.32 a	1.85 a	1615 a	81.0 b	19.0 b
2014	10.7 b	1.55	1.01 b	-	4.6 b	0.32	0.22 a	-	1.88 a	-	82.6 b	17.4 b
2016	11.8 c	2.17	1.76 d	3.00 c	9.2 c	0.72	0.57 b	0.989 b	2.86 c	3890 c	75.9 a	24.1 c
**ANOVA**												
Rootstock (R)	*	***	***	*	ns	ns	ns	ns	**	ns	ns	ns
Irrigation strategy (IS)	Ns	ns	ns	ns	ns	ns	ns	ns	ns	ns	ns	ns
Year	***	***	***	***	***	***	***	***	***	***	***	***
Interaction (R × IS)	ns	ns	ns	ns	ns	ns	ns	ns	ns	ns	ns	ns

ns, not significant; *, **, and *** indicate significant differences at the 0.05, 0.01, and 0.001 levels of probability, respectively. In each column and for each factor or interaction, different letters indicate significant differences according to Duncan’s multiple range test at the 95% confidence level.

**Table 6:** Matrix of the Pearson’s correlation coefficients obtained between the percentage distribution of cells by typology in the epidermis and hypodermis and the total anthocyanins and total grape phenolic compounds concentrations in the skins of Monastrell grapes. The data are from 2015.

	Epidermis					Hypodermis				
	Absence of Coloration (%)	Uniform Coloration (%)	Fine Granulation (%)	Small Spherical Inclusions (%)	Large Inclusions (%)	Absence of Coloration (%)	Uniform Coloration (%)	Fine Granulation (%)	Small Spherical Inclusions (%)	Large Inclusions (%)
Skin total anthocyanins (mg g^−1^ skin)	−0.27	0.37	−0.60	−0.37	−0.20	0.30	0.25	−0.39	−0.32	0.20
Skin total anthocyanins (mg berry^−1^)	−0.16	0.37	−0.53	−0.58	−0.22	0.21	0.32	−0.45	−0.22	0.27
Skin total anthocyanins (mg g^−1^ berry)	−0.26	0.58	−0.56	−0.69 *	−0.46	0.25	0.46	−0.53	−0.38	0.35
Skin total phenolic compounds (mg g^−1^ skin)	−0.53	0.59	−0.59	−0.32	−0.41	0.25	0.32	−0.43	−0.37	0.35
Skin total phenolic compounds (mg berry^−1^)	−0.37	0.72 *	−0.46	−0.70 *	−0.63 *	0.20	0.49	−0.54	−0.41	0.46
Skin total phenolic compounds (mg g^−1^ berry)	−0.14	0.55	−0.36	−0.78 **	−0.50	0.24	0.38	−0.50	−0.30	0.32
Berry total phenolic compounds (mg g^−1^ berry)	−0.49	0.76 *	−0.53	−0.57	−0.64 *	0.10	0.69 *	−0.55	−0.49	0.46
Berry total phenolic compounds (mg berry^−1^)	0.19	0.13	−0.05	0.23	0.44	0.85	0.09	0.11	0.43	0.23

* and ** indicate significant differences at, respectively, the 0.05 and 0.01 levels of probability.

**Table 7:** Carbohydrate composition of Monastrell grapes from 2014 to 2016, for five different rootstocks (140Ru, 1103P, 41B, 110R, and 161-49C) and two different irrigation strategies (PRI and RDI). Glucose, fructose, and sucrose (mg kg^−1^ berry); starch (mg glucose kg^−1^ berry).

Rootstock (R)		Glucose	Fructose	Sucrose	G+F+S	G/F	Starch
140Ru		90 abc	95 ab	9.6 b	192 ab	0.94	5.08 a
1103P		87 ab	90 a	8.3 a	186 a	0.95	4.79 a
41B		86 a	90 a	8.3 a	184 a	0.95	5.20 ab
110R		95 bc	96 ab	9.2 ab	198 ab	0.96	4.94 a
161-49C		99 c	102 b	8.0 a	209 b	0.95	5.61 b
**Irrigation strategy (IS)**							
PRI		93	95	8.9	195	0.95	5.21
RDI		90	94	8.5	193	0.95	5.04
Year							
2014		77 b	84 a	3.5 a	165 b	0.92	5.60 b
2015		69 a	83 a	4.1 a	152 a	0.82	5.26 b
2016		128 c	116 b	18.5 b	265 c	1.10	4.52 a
**R × IS**							
140Ru	PRI	94 abc	97 a	9.9	202 abc	0.94	5.39 bc
	RDI	85 ab	93 a	9.3	183 ab	0.94	4.78 ab
1103P	PRI	80 a	85 a	7.8	173 a	0.94	5.07 abc
	RDI	93 abc	96 a	8.9	199 ab	0.95	4.50 a
41B	PRI	85 ab	89 a	8.7	182 ab	0.95	4.76 ab
	RDI	87 ab	91 a	7.8	186 ab	0.95	5.64 cd
110R	PRI	95 bc	94 a	9.3	193 ab	0.95	4.67 ab
	RDI	96 bc	98 ab	9.1	203 bc	0.96	5.22 abc
161-49C	PRI	108 c	111 b	8.6	227 c	0.95	6.16 d
	RDI	90 ab	94 a	7.5	191 ab	0.94	5.06 abc
**ANOVA**							
R		*	*	*	*	ns	*
IS		ns	ns	ns	ns	ns	ns
Year		***	***	***	***	***	***
R × IS		*	*	ns	**	ns	***

ns, not significant; *, **, and *** indicate significant differences at the 0.05, 0.01, and 0.001 levels of probability, respectively. In each column and for each factor or interaction, different letters indicate significant differences according to Duncan’s multiple range test at the 95% confidence level.

**Table 8:** Must color parameters of the Monastrell berries, for five different rootstocks (140Ru, 1103P, 41B, 110R, and 161-49C) and two different irrigation strategies (PRI and RDI), from 2012 to 2016. The chromatic characteristics of the must (L*, a*, b*, C*, and h*) were measured in 2016.

Rootstock (R)		OD_620_	OD_520_	OD_420_	IC	Tone	L*	a*	b*	C*	h*
140Ru		0.53 a	1.98 a	1.98 a	4.49 a	1.04 d	22.8	15.2 a	3.2 ab	15.6 a	11.0 a
1103P		0.61 b	2.18 bc	2.22 b	5.02 b	1.03 d	23.0	16.2 ab	3.6 abc	16.7 ab	11.7 ab
41B		0.52 a	2.16 b	2.00 a	4.68 a	0.94 b	23.3	19.2 c	4.8 c	20.1 c	13.5 b
110R		0.62 b	2.32 cd	2.27 b	5.20 b	0.99 c	22.5	15.0 a	3.0 a	15.3 a	10.9 a
161-49C		0.55 a	2.44 d	2.12 a	5.16 b	0.88 a	22.9	18.4 bc	4.4 bc	18.9 bc	12.8 ab
**Irrigation strategy (IS)**											
PRI		0.53	2.20	2.03	4.77	0.95	23.1	18.2	4.3	18.7	12.8
RDI		0.60	2.25	2.20	5.05	1.00	22.8	15.4	3.3	15.9	11.1
**Year**											
2012		0.53 c	1.97 b	1.98 b	4.47 b	1.00 c	-	-	-	-	-
2013		0.33 -a	1.57 a	1.51 a	3.41 a	0.99 c	-	-	-	-	-
2014		0.82 e	2.42 d	2.83 d	6.07 c	1.18 d	-	-	-	-	-
2015		0.42 b	2.18 c	1.86 b	4.46 b	0.90 b	-	-	-	-	-
2016		0.72 d	2.94 e	2.38 c	6.04 c	0.81 a	22.9	16.8	3.8	17.3	12.0
**Interaction (R** **× IS)**											
140Ru	PRI	0.49 a	2.00 ab	1.90	4.39 a	1.00 bc	23.0	18.0	4.3	18.6	12.8
	RDI	0.57 ab	1.96 a	2.06	4.59 ab	1.08 d	22.6	12.4	2.1	12.6	9.2
1103P	PRI	0.55 ab	2.08 abc	2.05	4.68 abc	0.99 b	23.5	19.0	4.6	19.6	13.3
	RDI	0.67 c	2.28 bc	2.40	5.35 d	1.07 cd	22.6	13.5	2.7	13.8	10.0
41B	PRI	0.48 a	2.05 ab	1.90	4.43 ab	0.94 b	23.5	19.7	5.0	20.4	13.7
	RDI	0.55 ab	2.27 bc	2.10	4.93 bcd	0.94 b	23.1	18.6	4.5	19.8	13.3
110R	PRI	0.61 bc	2.26 abc	2.23	5.10 cd	1.00 bc	22.6	16.1	3.4	16.4	11.5
	RDI	0.63 bc	2.37 cd	2.31	5.31 d	0.98 b	22.4	14.0	2.7	14.2	10.3
161-49C	PRI	0.54 ab	2.62 d	2.09	5.24 d	0.83 a	22.6	18.3	4.1	18.7	12.5
	RDI	0.57 ab	2.37 abcc	2.15	5.08 abc	0.93 b	23.2	18.5	4.7	19.1	13.0
**ANOVA**											
R		***	***	***	***	***	ns	*	*	*	*
IS		***	ns	***	*	***	ns	**	*	**	*
Year		***	***	***	***	***	-	-	-	-	-
R × IS		***	**	ns	*	**	ns	ns	ns	ns	ns

ns, not significant; *, **, and *** indicate significant differences at the 0.05, 0.01, and 0.001 levels of probability, respectively. In each column and for each factor or interaction, different letters indicate significant differences according to Duncan’s multiple range test at the 95% confidence level.

**Table 9:** Matrix of Pearson’s correlation coefficients obtained between the berry phenolic maturity and wine quality parameters and the skin polyphenolic distribution and skin morphological characteristics (data from 2015). EA (extractable anthocyanins), TA (total anthocyanins), SM index (seed maturity index), QI_phenolic_ (phenolic berry quality index), CI (color intensity), TPI (total phenolic index), and QI_wine_ (wine quality index).

	Grape Quality Parameters					Wine Quality Parameters				
	Polyphenols	EA	TA	SM index	QI_phenolic_	CI	TPI	Anthocyanins	Tannins	QI_wine_
Skin cell size	−0.10	−0.29	−0.33	0.30	−0.48	−0.60	−0.09	−0.51	0.38	0.05
Epidermis cell size	0.02	−0.21	−0.32	0.49	−0.24	−0.67 *	0.04	−0.46	0.41	0.10
Hypodermis cell size	−0.08	−0.25	−0.29	0.26	−0.46	−0.58	−0.11	−0.53	0.37	0.02
Pulp cell size	0.16	−0.13	−0.02	0.21	0.07	0.03	−0.01	0.16	0.15	0.07
Hypodermis cell size/epidermis cell size	−0.23	−0.02	0.12	−0.26	−0.31	0.37	−0.26	0.08	−0.32	−0.23
Pulp cell size/skin cell size	0.33	0.43	0.49	−0.22	0.53	0.55	−0.09	0.41	−0.24	−0.21
Skin thickness	0.10	0.09	0.00	0.07	−0.14	−0.46	−0.09	−0.54	0.15	−0.02
Epidermis thickness	0.03	−0.17	−0.18	0.38	−0.16	−0.86 **	−0.06	−0.54	0.15	−0.02
Hypodermis thickness	0.10	0.12	0.02	0.04	−0.13	−0.40	−0.09	−0.48	0.27	−0.17
Skin cell wall thickness	0.08	0.20	0.13	−0.06	0.02	−0.29	−0.34	−0.68 *	0.43	−0.67 *
Epidermis cell wall thickness	0.49	0.09	0.08	0.63	0.36	−0.52	−0.24	−0.11	−0.15	−0.08
Hypodermis cell wall thickness	−0.01	0.21	0.14	−0.21	−0.04	−0.17	−0.29	−0.66 *	0.46	−0.65 *
Cuticle thickness	−0.41	−0.01	0.10	0.76 **	0.15	−0.02	0.22	−0.19	−0.24	−0.27
Epidermal cells without coloration	0.02	−0.21	−0.30	0.49	−0.27	−0.43	0.10	−0.02	−0.06	0.46
Epidermal cells with uniform coloration	−0.07	0.16	0.23	−0.46	−0.01	0.61	−0.18	0.25	−0.23	−0.25
Epidermal cells with fine granules	−0.02	−0.46	−0.55	0.65 *	0.08	−0.23	0.45	0.29	0.08	0.66 *
Epidermal cells with small spherical inclusions	0.33	0.05	0.03	0.36	0.67 *	−0.25	0.22	−0.02	0.37	0.10
Epidermal cells with large inclusions	−0.02	−0.07	−0.11	0.18	−0.03	−0.58	0.10	−0.45	0.30	−0.05
Hypodermal cells without coloration	−0.34	−0.09	0.02	−0.49	−0.66 *	−0.05	−0.19	−0.27	−0.18	−0.27
Hypodermal cells with uniform coloration	0.18	0.46	0.41	−0.37	0.34	0.86 **	0.06	0.50	0.06	0.12
Hypodermal cells with fine granules	0.23	−0.23	−0.28	0.69 *	0.32	−0.21	0.29	0.33	0.06	0.60
Hypodermal cells with small spherical inclusions	0.16	−0.05	−0.13	0.45	−0.03	−0.76 *	−0.06	−0.48	0.05	−0.24
Hypodermal cells with large inclusions	−0.32	−0.05	0.01	−0.43	0.23	0.16	−0.22	−0.33	0.10	−0.59

* and ** indicate significant differences at, respectively, the 0.05 and 0.01 levels of probability.

**Table 10:** Matrix of Pearson’s correlation coefficients obtained between the color and phenolic parameters of the must and color intensity (CI) of the wine and the berry total phenolic compounds (TPC) and total anthocyanins concentrations. The must data are from four years (2012, 2013, 2014, 2016) and the wine data are from three years (2014–2016). OD (optical density), AE index (anthocyanin extractability index), and SM Index (seed maturity index).

	Must										Wine
	OD_620_	OD_520_	OD_420_	CI	Tone	Polyphenols Content (mg L^−1^)	Extractable Anthocyanins (mg L^−1^)	Total Anthocyanins (mg L^−1^)	AE Index	SM Index	CI
Total anthocyanins (mg g^−1^ skin)	0.33 *	0.30	0.32 *	0.33 *	0.04	0.47 **	0.89 ***	0.91 ***	−0.49 **	−0.87 ***	0.09
Total anthocyanins (µg g^−1^ berry)	0.36 *	0.56 ***	0.27	0.43 **	−0.45 **	0.07	0.64 ***	0.74 ***	−0.12	−0.83 ***	0.51 *
Total anthocyanins (µg g^−1^ must)	0.79 ***	0.80 ***	0.75 ***	0.81 ***	−0.50 **	0.15	0.56 ***	0.63 ***	−0.22	−0.74 ***	−0.43
Skin TPC (mg g^−1^ skin)	0.46 **	0.51 ***	0.41 **	0.49 **	−0.19	0.39 *	0.87 ***	0.89 ***	−0.46 **	−0.92 ***	0.75 ***
Skin TPC (µg g^−1^ berry)	0.41 **	0.64 ***	0.30	0.49 **	−0.51 ***	−0.02	0.52 ***	0.62 ***	−0.06	−0.75 ***	0.57 **
Skin TPC (µg g^−1^ must)	0.83 ***	0.85 ***	0.78 ***	0.85 ***	−0.54 **	0.06	0.44 *	0.50 **	−0.16	−0.64 ***	−0.46
Seed TPC (mg g^−1^ seed)	0.39 *	0.72 ***	0.28	0.51 ***	−0.56 ***	−0.23	0.03	0.14	0.17	−0.28	0.83 ***
Seed TPC (µg g^−1^ berry)	0.38 *	0.73 ***	0.26	0.51 ***	−0.63 ***	−0.30	0.01	0.13	0.22	−0.29	0.79 ***
Seed TPC (µg g^−1^ must)	0.68 ***	0.84 ***	0.61 ***	0.76 ***	−0.69 ***	−0.26	−0.02	0.07	0.15	−0.20	0.38
Total TPC (µg g^−1^ berry)	0.43 **	0.72 ***	0.31	0.53 ***	−0.58 ***	−0.11	0.40 *	0.51 ***	0.03	−0.66 ***	0.66 **
Total TPC (µg g^−1^ must)	0.22	0.54 ***	0.10	0.32 *	−0.64 ***	−0.04	0.31	0.39 *	−0.07	0.53 **	0.77 ***

*, **, and *** indicate significant differences at the 0.05, 0.01, and 0.001 levels of probability, respectively.

**Table 11:** Matrix of Pearson’s correlation coefficients obtained between the wine quality parameters and the phenolic quality of Monastrell berries. The data are from three years (2014–2016). AE index (anthocyanin extractability index), SM Index (seed maturity index), TPI (total phenolic index), and QI_wine_ (wine quality index).

			Wine	
Berry	TPI	Tannins	Anthocyanins	QI_wine_
Polyphenols content	−0.14	−0.26	0.44 *	−0.22
Extractable anthocyanins	0.27	−0.07	0.83 ***	0.22
Total anthocyanins	0.36 *	0.03	0.83 ***	0.30
AE Index	0.17	0.40 *	−0.48 **	0.15
SM Index	−0.38 *	0.01	−0.81 ***	−0.37 *

*, **, and *** indicate significant differences at the 0.05, 0.01, and 0.001 levels of probability, respectively.

## Supplementary Materials

The following are available online at https://www.mdpi.com/article/10.3390/plants10122585/s1, Table S1: berry, pulp, skin, and seed parameters evaluated by tasters and the scores given to evaluate the taste and the sensory parameters of Monastrell grapes, Table S2: the scores obtained in the tasting and sensory analysis of the berries, for five different rootstocks (140Ru, 1103P, 41B, 110R, and 161-49C) and two different irrigation strategies (PRI and RDI), in 2015, Table S3: matrix of Pearson’s correlation coefficients obtained between physico-chemical parameters of berry quality and the scores obtained in the tasting and sensory analysis of the berries in 2015.
