# Supplementary material for: Changes in Berry Tissues in Monastrell Grapevines Grafted on Different Rootstocks and Their Relationship with Berry and Wine Phenolic Content
Source: Plants (Basel). 2021 Nov 25;10(12):2585. doi: 10.3390/plants10122585 (PMC8705433; doi:10.3390/plants10122585)
Supplement: Supplementary file 1 [file plants-10-02585-s001.zip › plants-1442189-supplementary.pdf]

SUPPORTING INFORMATION

Table S1. Berry, pulp, skin, and seed parameters evaluated by tasters and the scores given to evaluate the taste and the sensory parameters of Monastrell grapes.

BERRY	1	2	3	4
Crushability	Hard, it breaks only witl strong pressure	It deforms, slightly elastic	It deforms slightly but returns to the initial forr	Weak grain
Easy of peeling	Strongly adhering pedicel	Attached pedicel that comes off easily	Pedicel that comes off relatively easily	Pedicel that comes off very easily
**PULP**	**1**	**2**	**3**	**4**
Adherence	Pulp strongly adhering to the skin	Layer of the attached and visible pulp	Fine stratum of the pulp poorly visible	No release of juice when chewing
Sweetness	Little sweetness	Moderately sweet	Sweet	Very sweet
Acidity	Low	Mildly acidic	Acidic	Very acidic
**SKIN**	**1**	**2**	**3**	**4**
Maceration	Very hard	Moderately hard	Easily crushed	Fragile
Tannic Intensit	Very low	Quite low	Moderately high	High
Astringency	Very low	Quite low	Moderately low	High
**SEEDS**	**1**	**2**	**3**	**4**
Color	White or greenish-yellow	Greenish-brown	Dark gray	Dark brown
Crushability	Hard	First softness, then hard seed	It breaks quite easily	Fragile and crispy
Aroma	Unable to be tasted	Green or herbaceous	Toasted	Roasted

Table S2. The scores obtained in the tasting and sensory analysis of the berries, for five different rootstocks (140Ru, 1103P, 41B, 110R, and 161-49C) and two different irrigation strategies (PRI and RDI), in 2015.

		Visual/tactile examination of berries		Tasting of the pulp			Tasting of the skin			Seeds			Maturity
**Rootstock (R)**		Crushability	Peeling	Adherence	Sweetness	Acidity	Maceration	Tannic intensity	Astringency	Color	Crushability	Aroma	Total Score
140Ru		2.2	2.8	2.3	2.5	1.7	1.7	2.8	2.6	2.4	2.4	2.7	2.5
1103P		2.8	2.4	2.4	2.2	1.5	2.1	2.9	2.5	2.3	2.1	3.0	2.5
41B		2.3	2.5	2.7	2.8	1.1	2.2	3.1	2.8	2.4	2.2	3.0	2.7
110R		2.3	2.5	2.4	2.3	1.2	2.4	2.5	2.3	3.0	2.6	3.1	2.6
161-49C		2.1	3.0	2.7	2.9	1.6	2.5	2.5	2.4	2.5	2.1	2.7	2.5
**Irrigation system (IS)**													
PRI		2.4	2.7	2.4	2.5	1.4	2.2	2.7	2.4	2.4	2.4	2.9	2.5
RDI		2.3	2.6	2.6	2.6	1.4	2.2	2.8	2.6	2.7	2.2	2.9	2.6
**R × IS**													
140RU	PRD	2.0	2.6	2.0a	2.6	2.0	1.6	2.8	2.4	2.0	2.2	2.6	2.3
	RDI	2.4	3.0	2.6abc	2.4	1.4	1.8	2.8	2.8	2.8	2.6	2.8	2.7
1103P	PRD	2.6	2.8	2.6abc	2.0	1.4	2.0	2.8	2.4	2.2	2.4	2.8	2.5
	RDI	3.0	2.0	2.2ab	2.4	1.6	2.2	3.0	2.6	2.4	1.8	3.2	2.5
41B	PRD	2.4	2.6	2.8bc	2.8	1.0	2.2	2.8	2.8	2.6	2.6	3.0	2.7
	RDI	2.2	2.4	2.6abc	2.8	1.2	2.2	3.4	2.8	2.2	1.8	3.0	2.6
110R	PRD	2.4	2.4	2.4ab	2.2	1.2	2.6	2.8	2.4	2.8	2.6	3.4	2.6
	RDI	2.2	2.6	2.4ab	2.4	1.2	2.2	2.2	2.2	3.2	2.6	2.8	2.6
161-49C	PRD	2.6	3.0	2.2ab	3.0	1.6	2.6	2.2	2.0	2.2	2.0	2.8	2.5
	RDI	1.6	3.0	3.2c	2.8	1.6	2.4	2.8	2.8	2.8	2.2	2.6	2.6
**ANOVA**													
R		ns	ns	ns	ns	ns	ns	ns	ns	ns	ns	ns	ns
IS		ns	ns	ns	ns	ns	ns	ns	ns	ns	ns	ns	ns
R × IS		ns	ns	*	ns	ns	ns	ns	ns	ns	ns	ns	ns

ns, not significant; * indicates significant differences at the 0.05 level of probability. In each column and for each factor or interaction, different letters indicate significant differences according to Duncan's multiple range test at the 95% confidence level.

Table S3. Matrix of Pearson´s correlation coefficients obtained between physico-chemical parameters of berry quality and the scores obtained in the tasting and sensory analysis of the berries in 2015.

	Crushability	Peeling	Sweetness	Skin maceration	Tannic intensity	Astringency	Seed Crushability
Polyphenols	0.30	-0.19	0.31	0.78**	0.03	-0.19	-0.37
EA	0.41	0.11	0.28	0.76*	-0.49	-0.67*	-0.19
TA	0.26	0.20	0.44	0.69*	-0.56	-0.70*	-0.18
Acidity	0.32	-0.03	-0.69*	0.01	-0.15	-0.35	0.05
Tartaric acid	-0.18	0.76*	-0.32	-0.26	-0.38	-0.16	0.41
Malic acid	-0.64*	0.51	0.50	-0.25	-0.15	0.21	0.09
OD_{520}	0.39	-0.15	-0.79**	-0.27	0.18	-0.13	0.17
IC	0.35	-0.18	-0.73*	-0.26	0.25	-0.08	0.10
Tone	-0.40	0.23	0.80**	0.39	-0.21	0.01	-0.36
QI_{total}	0.00	0.41	0.45	0.50	-0.45	-0.58	-0.40
$QI_{technol}$	-0.28	0.57	0.54	0.39	-0.47	-0.45	-0.26
$QI_{phenolic}$	0.35	0.11	0.25	0.55	-0.34	-0.65*	-0.52
Amino acids	-0.37	0.67*	0.15	0.34	-0.70*	-0.49	0.17
Resveratrol	-0.39	0.59	0.55	0.42	-0.65*	-0.30	0.11
Glucose	-0.02	-0.08	0.48	0.69*	0.05	-0.25	-0.70*
Fructose	-0.08	0.04	0.48	0.69*	0.00	-0.29	-0.63
G+F+S	-0.05	-0.02	0.48	0.69*	0.03	-0.27	-0.66*

* and ** indicate significant differences at the 0.05 and 0.01 levels of probability, respectively.
